# Disease progression in patients with single, large-scale mitochondrial DNA deletions

**DOI:** 10.1093/brain/awt321

**Published:** 2013-11-23

**Authors:** John P. Grady, Georgia Campbell, Thiloka Ratnaike, Emma L. Blakely, Gavin Falkous, Victoria Nesbitt, Andrew M. Schaefer, Richard J. McNally, Grainne S. Gorman, Robert W. Taylor, Doug M. Turnbull, Robert McFarland

**Affiliations:** 1 Wellcome Trust Centre for Mitochondrial Research, Institute for Ageing and Health, Newcastle University, Newcastle upon Tyne, UK; 2 Institute of Health and Society, Newcastle University, Newcastle upon Tyne, UK

**Keywords:** mitochondrial diseases, mitochondrial DNA deletion, disease progression

## Abstract

Single, large-scale deletions of mitochondrial DNA are a common cause of mitochondrial disease and cause a broad phenotypic spectrum ranging from mild myopathy to devastating multi-system syndromes such as Kearns-Sayre syndrome. Studies to date have been inconsistent on the value of putative predictors of clinical phenotype and disease progression such as mutation load and the size or location of the deletion. Using a cohort of 87 patients with single, large-scale mitochondrial DNA deletions we demonstrate that a variety of outcome measures such as COX-deficient fibre density, age-at-onset of symptoms and progression of disease burden, as measured by the Newcastle Mitochondrial Disease Adult Scale, are significantly (*P* < 0.05) correlated with the size of the deletion, the deletion heteroplasmy level in skeletal muscle, and the location of the deletion within the genome. We validate these findings with re-analysis of 256 cases from published data and clarify the previously conflicting information of the value of these predictors, identifying that multiple regression analysis is necessary to understand the effect of these interrelated predictors. Furthermore, we have used mixed modelling techniques to model the progression of disease according to these predictors, allowing a better understanding of the progression over time of this strikingly variable disease. In this way we have developed a new paradigm in clinical mitochondrial disease assessment and management that sidesteps the perennial difficulty of ascribing a discrete clinical phenotype to a broad multi-dimensional and progressive spectrum of disease, establishing a framework to allow better understanding of disease progression.

## Abstract (precis)

Single, large-scale deletions of mitochondrial DNA are an important cause of mitochondrial disease, with a broad phenotypic spectrum. Grady *et al.* report that disease severity and progression are correlated with the size of the deletion, its location within the genome, and the deletion heteroplasmy level in skeletal muscle.

## Introduction

Mitochondrial genetic defects are an important cause of neurological disease and are a result of mutations of either the nuclear or mitochondrial genome (McFarland *et al.*, 2010; Schon *et al.*, 2012). The ability to understand and predict the progression of mitochondrial disease is of great clinical importance to give patients guidance about their risk of developing symptoms and enabling clinicians to provide optimum patient care. However, to date, fundamental understanding of the factors governing mitochondrial disease progression is limited and studies are often contradictory in their findings. We therefore chose to concentrate on one of the most common mitochondrial diseases: patients with single, large-scale mitochondrial DNA deletions.

Characterization of the phenotypic presentation of single, large-scale mitochondrial DNA disease is traditionally divided into three main presentations (Schon *et al.*, 2012): Kearns-Sayre syndrome (Kearns and Sayre, 1958), a multi-system childhood or teenage-onset syndrome; chronic progressive external ophthalmoplegia (CPEO; Moraes *et al.*, 1989), a milder presentation involving principally weakness of the extraocular muscles with ptosis, but often associated with more widespread muscular weakness; and Pearson Syndrome (Pearson *et al.*, 1979), an often fatal infantile-onset syndrome characterized by sideroblastic anaemia and exocrine pancreatic dysfunction, which may develop into Kearns-Sayre syndrome in those who survive infancy. However, as many studies attempting to unpick phenotypic presentation have noted, these divisions are somewhat imprecise and misrepresent the true spectrum of single mitochondrial DNA deletion-related disease; patients denoted CPEO are often subdivided into groups such as ‘classic CPEO’ at the mild end of the spectrum, ‘severe CPEO’ or ‘CPEO+’, and ‘partial Kearns-Sayre syndrome’ or ‘CPEO + multisystem’ (Auré *et al.*, 2007).

Early investigations of single, large scale mitochondrial DNA deletion disease found little connection between mitochondrial genetics, biochemical defects and clinical phenotype (Zeviani *et al.*, 1988; Holt *et al.*, 1989; Moraes *et al.*, 1989; Rotig *et al.*, 1995). More recent studies are rather contradictory. Skeletal muscle heteroplasmy is variously reported as non-predictive of either phenotype or age at onset (Yamashita *et al.*, 2008), predictive of onset but not phenotype (López-Gallardo *et al.*, 2009), or predictive of both to a limited extent (Sadikovic *et al.*, 2010). Mitochondrial DNA deletion size is inconsistently reported as either predictive of phenotype and disease severity (Yamashita *et al.*, 2008) or the contrary (López-Gallardo *et al.*, 2009). Interestingly, two recent studies found that the location of the mitochondrial DNA deletion was predictive of phenotype or age at onset to an extent, though with contrary conclusions. It has been reported that mitochondrial DNA deletions including one of the three mitochondrial DNA-encoded structural components of cytochrome *c* oxidase (COX) (*MT-CO1*, *MT-CO2* or *MT-CO3* genes) and complex V (*MT-ATP6* or *MT-ATP8* genes) had significantly earlier onset (Yamashita *et al.*, 2008) although other studies have found that deletion of the mitochondrial cytochrome *b* (*MT-CYB*) gene was significantly associated with a severe Kearns-Sayre syndrome phenotype (López-Gallardo *et al.*, 2009).

Researchers have attempted to better segregate patients into groups that are easier to analyse, for instance by the presence of cerebellar involvement (Auré *et al.*, 2007) or purely myopathic symptoms (López-Gallardo *et al.*, 2009). However, for mitochondrial genetic disorders, the disease spectrum is multidimensional, with a wide range of potential systems involved, each to varying degrees of severity. This severely hampers any attempt to discretely classify the disease. The Newcastle Mitochondrial Disease Adult Scale (NMDAS) was published as a semi-quantitative rating scale to monitor mitochondrial disease (Schaefer *et al.*, 2006). It is a clinically validated tool that has been extensively used both at our centre (Apabhai *et al.*, 2011; Bates *et al.*, 2013; Lax *et al.*, 2012) and other specialist mitochondrial centres (de Laat *et al.*, 2012; Enns *et al.*, 2012; Orsucci *et al.*, 2012; Yatsuga *et al.*, 2012; Kornblum *et al.*, 2013; Mancuso *et al.*, 2013). The NMDAS permits quantitative analysis of both general and system-specific disease progression and has already been used in assessment of clinical progression in patients with the m.3243A>G mitochondrial DNA mutation, although this was not a longitudinal study (Whittaker *et al.*, 2009). Linear mixed modelling has been widely used to understand disease progression in other neurodegenerative conditions including dementia (Tornatore and Grant, 2002; Hassing *et al.*, 2004; Galvin Je and *et al.*, 2005; Johnson *et al.*, 2009; Knopman *et al.*, 2009; den Heijer *et al.*, 2010), Parkinson’s disease (Nandhagopal *et al.*, 2009; Dobkin *et al.*, 2011; Johnson and Galvin, 2011; Vu *et al.*, 2012) and multiple sclerosis (Meier *et al.*, 2007), and NMDAS facilitates such longitudinal modelling of mitochondrial disease progression.

In this study, we used repeated measures mixed modelling with collated NMDAS data for a large patient cohort to test our hypothesis that mitochondrial DNA heteroplasmy levels and mitochondrial DNA deletion size and location are predictive of single, large-scale mitochondrial DNA deletion disease progression. We also applied multiple linear regression analyses to examine the relationship between these predictors and outcome measures such as age at onset, clinical phenotype and severity of the biochemical defect (as determined by COX-deficient fibre density) in both our cohort and a meta-analysis of previously published data.

## Materials and methods

### Newcastle patient cohort

All patients were investigated by the NHS Highly Specialized Service for Rare Mitochondrial Disorders in Newcastle upon Tyne. The majority of the patients (55 of 87) were regularly followed in our clinic at 6 or 12 monthly intervals, assessed using the NMDAS (Schaefer *et al.*, 2006) and recruited to the MRC Mitochondrial Disease Patient Cohort UK. The remaining 32 patients include individuals not seen in our clinic and for whom we have limited clinical information or for whom we do not have NMDAS data available. For some of these patients we have been able to ascertain disease onset from hospital records. A full listing of the clinical and molecular characteristics of the patient cohort can be found in Supplementary Table 1.

Phenotypic analysis compared patients in two groups, those classified as ‘CPEO’ or ‘CPEO + myopathy’ (54 patients) and those classified as ‘Kearns-Sayre syndrome’ (nine patients). For other analyses, we used all the patients in our cohort that had the appropriate information available, as listed in Table 1.

**Table 1:** Summary of Newcastle patient cohort and available data in literature

	With NMDAS	Without NMDAS	Our cohort (Total)	Meta-analysis
Number of patients with mitochondrial DNA deletion size and muscle mitochondrial DNA heteroplasmy data	55	32	**87**	256
Number of patients with mitochondrial DNA breakpoints identified	52	31	**83**	184
Number of patient with age at onset data	52	8	**60**	117
Number of patients with age at onset data and mitochondrial DNA breakpoints identified	49	7	**56**	83
Number of patients with COX-deficient fibre density data and mitochondrial DNA breakpoints identified	49	23	**72**	40
Number of patients presenting with a CPEO, CPEO + myopathy or KSS phenotype	36	27	**63**	205
Number of patients presenting with a CPEO, CPEO + myopathy or KSS phenotype and mitochondrial DNA breakpoints identified	35	26	**61**	149

All patients have skeletal muscle heteroplasmy and deletion size data available. Where gene location is under investigation the sub-cohort of patients with identified mitochondrial DNA breakpoints is used; where location is not under consideration, the larger cohort with mitochondrial DNA deletion size is used. The small cohort of patients with phenotype data reflects the fact that patients with a multisystem phenotype are excluded from the phenotype analysis, so this number is restricted to patients with CPEO, CPEO + myopathy, or Kearns-Sayre (KSS) syndrome. The patients with NMDAS data available had a median of five assessments (interquartile range 5).

### Meta-analysis

The foundation of our meta-analysis is a previous meta-analysis by López-Gallardo *et al.* (2009), which includes data on patient age at disease-onset, patient age at biopsy, clinical phenotype, mitochondrial DNA deletion load (heteroplasmy) in muscle, mitochondrial DNA deletion size and specific mitochondrial DNA deletion breakpoints. We used the data as published by López-Gallardo *et al.* (2009) except where review of the literature identified differences between the published data and that used by this group (three cases) or if there was inconsistency between the reported mitochondrial DNA deletion size and location of the breakpoints (one case). Additionally, we eliminated those cases where the reported mitochondrial DNA deletion was characterized by restriction endonuclease digests and as such there was uncertainty as to whether specific genes under scrutiny (*MT-CO* or *MT-CYB*) were deleted (seven cases) or there were other reported mutations in the same patient (nine cases). We also excluded one patient with a highly unusual mitochondrial DNA deletion in the minor arc of the mitochondrial genome. Additional cases were obtained from other publications identified through PubMed not included in the study by López-Gallardo *et al.* (2009) (Johns *et al.*, 1989; Mita *et al.*, 1989; Larsson and Holme, 1992; Oldfors *et al.*, 1992; Ishikawa *et al.*, 2000; Gellerich *et al.*, 2002; Jacobs *et al.*, 2004; Sadikovic *et al.*, 2010). These data are detailed in Supplementary Table 2. All amendments and exclusions are listed in Supplementary Table 3. We do not include our own cohort in the meta-analysis to ensure sample independence.

For the phenotypic analysis, patients previously classified by López-Gallardo and colleagues (2009) as ‘CPEO without non-muscular signs’ were combined with patients classified ‘CPEO’ or ‘CPEO + myopathy’ from the other literature (101 cases in total) and compared with patients classified as ‘Kearns-Sayre syndrome’ from all sources (104 cases in total). For other analyses, we used all the patients in the meta-analysis that had the appropriate information available, as listed in Table 1.

### Muscle biopsy histochemistry data

Sequential COX/succinate dehydrogenase histochemical reactions were performed on skeletal muscle sections following standardized protocols (Old and Johnson, 1989). The extent of the COX deficiency (percentage of COX-deficient fibres) was determined by counting all fibres in a section (minimum 200 fibres) and performed by a single researcher to ensure data were recorded consistently across the entire cohort.

### Determination of level of mitochondrial DNA deletion

All molecular analyses were performed using total skeletal muscle DNA extracted using standard protocols. A validated, multiplex real-time PCR (quantitative PCR) *MTND1*/*MTND4* assay was used to quantify mitochondrial DNA deletion levels in muscle homogenates (He *et al.*, 2002; Krishnan *et al.*, 2007).

### Determination of mitochondrial DNA deletion size and location

Long-range PCR was used to amplify ∼9.5 kb region of the mitochondrial genome across the major arc using a single primer set corresponding to nucleotides 6378–15896 (GenBank Accession number: NC_012920.1). PCR reactions used ∼100 ng of DNA that was added to PCR mastermix [distilled H_2_O, LA Taq buffer (TaKaRa), 10 mM dNTPs, 20 mM forward and reverse primers and 2.5 units of LA Taq enzyme (TaKaRa)] to a total volume of 50 μl and subjected to the following cycling conditions: 94°C for 1 min; 35 cycles of 94°C for 30 s, 58°C for 30 s and 68°C for 11 min; final extension of 72°C for 10 min. Amplified products were separated through a 0.7% agarose gel, using a 1 kb DNA ladder to estimate product size and determine mitochondrial DNA deletion sizes.

Long-range PCR products were further assessed by restriction digests to map the precise location of the mitochondrial DNA deletion breakpoints (Khrapko *et al.*, 1999). PCR amplimers (5 µl) were digested to a series of DNA fragments of known length and position within the mitochondrial genome using restriction enzymes XhoI, BamHI, XcmI and DraI (New England Biolabs) before separation through a 0.7% agarose gel. The size of restriction products allows the location of the mitochondrial DNA deletion within the genome to be estimated, guiding the choice of appropriate sequencing primers (Supplementary Table 4) to characterize mitochondrial DNA deletion breakpoints by Sanger sequencing (BigDye® Terminator chemistries using an ABI 3130xl Genetic Analyser; Applied Biosystems).

### Statistical analyses

To investigate the relationship between the variables being tested, we used Box-Cox to stabilize the variance of the dependent variables (Box and Cox, 1964) and to identify optimal transformations of the variables that satisfy normality assumptions, enabling use of parametric tests. For basic analyses, a single summary data point was determined for each patient using the mean NMDAS score and mean age at assessment. Longitudinal modelling was conducted using SAS PROC MIXED in accordance with published guidelines (Singer, 1998; Diggle *et al.*, 2002). Data covariance structure was modelled using a spatial power structure (Moser, 2004). Polynomial terms of time up to cubed (time^3^) were included. Models were compared using the Akaike Information Criterion (Akaike, 1974; Kuha, 2004). Residual and influence diagnostics were conducted to validate model assumptions and verify model stability. SAS version 9.2 was used throughout. For predetermined hypothesis testing, significance was determined at *P* < 0.05, high significance at *P* < 0.0001. For multiple regression, we report the standardized coefficient (b) (standardized to have unit variance) and significance value (*P*-value) for each parameter estimate, together with the number of subjects (*n*) and the coefficient of determination (R^2^) for the overall regression. For simple linear regression we report *n*, the correlation coefficient (r) and the *P*-value. For multiple logistic regression we report *n*, and the standardized coefficient and *P*-value for each parameter estimate. For simple logistic regression we report *n*, the odds ratio for the effect of the dependent variable, and the *P*-value.

## Results

### Putative predictors of disease burden and progression are intercorrelated

**Figure 1:**
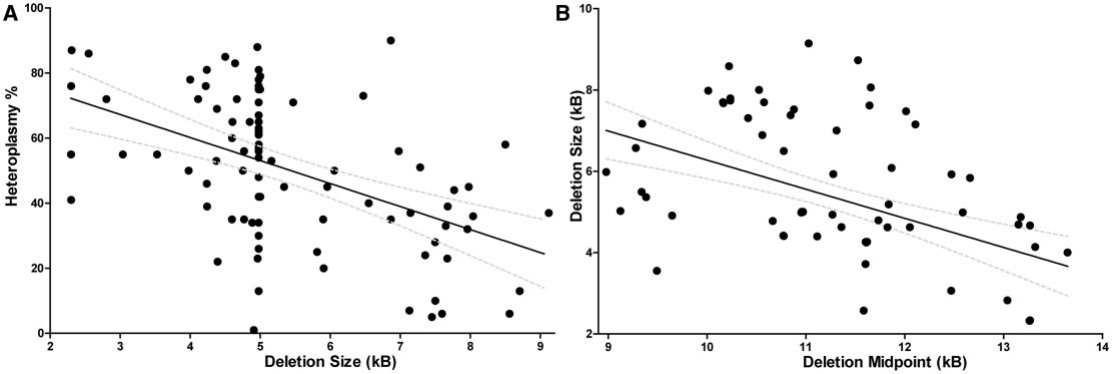
Putative predictors of disease progression are intercorrelated. (**A**) Skeletal muscle heteroplasmy is negatively correlated with mitochondrial DNA deletion size in our cohort. *n* = 87, r = −0.49, *P* < 0.0001. 95% CI is shown. The dense cluster of points around 5.0kb represents the cohort of patients with the common 4977 bp mitochondrial DNA deletion. (**B**) Mitochondrial DNA deletion size is negatively correlated with the location of the mitochondrial DNA deletion midpoint in our cohort. *n* = 83, r = −0.48, *P* < 0.0001. 95% CI is shown.

We began our investigations by looking at the relationships between the putative predictors of disease progression. In our patient cohort we observed a strong negative linear correlation between the level of deleted mitochondrial DNA compared with wild-type (i.e. mitochondrial DNA heteroplasmy) and mitochondrial DNA deletion size (*n* = 87, r = −0.49, *P* < 0.0001) (Fig. 1A), an observation confirmed in the meta-analysis (*n* = 256, r = −0.18, *P* = 0.0032). Mitochondrial DNA deletion size and location were also highly significantly correlated in our cohort (*n* = 83, r = −0.48, *P* < 0.0001; Fig. 1B), again confirmed by the meta-analysis (*n* = 184, r = −0.29, *P* < 0.0001). Highly significant correlations were also found between mitochondrial DNA heteroplasmy, mitochondrial DNA deletion size, and the two proposed genetic loci of interest (*MT-CO* and *MT-CYB* genes) that were identified in previous literature (Yamashita *et al.*, 2008; López-Gallardo *et al.*, 2009) (Table 2).

**Table 2:** Intercorrelations between putative predictors of disease burden and progression

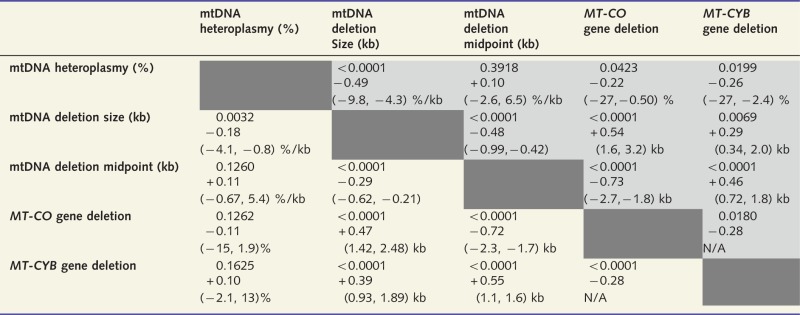

Shaded cells (upper right) show for our cohort *P*-values, correlation coefficients, and 95% confidence intervals for linear regression gradient or estimated difference due to specific mitochondrial DNA (mtDNA) gene deletion; unshaded cells (bottom left) show the same for the meta-analysis. Units for 95% confidence intervals identify *y* and *x* for linear regression, except in the case of mitochondrial DNA deletion size (kb) versus mitochondrial DNA deletion midpoint (kb). The strongest correlations in each data set are between mitochondrial DNA deletion midpoint or size and *MT-CO* gene deletion; larger mitochondrial DNA deletions tend to include *MT-CO* genes. *MT-CYB* deletion is also associated with larger mitochondrial DNA deletion size and mitochondrial DNA deletions. The same trends are seen in all cases in our cohort and the meta-analysis, excepting that in our cohort *MT-CYB* gene deletion is significantly associated with lower mitochondrial DNA heteroplasmy, whereas in the meta-analysis there is a non-significant trend relating *MT-CYB* gene deletion with higher mitochondrial DNA heteroplasmy levels.

### Age at onset, clinical phenotype and NMDAS progression are correlated with muscle heteroplasmy and mitochondrial DNA deletion size

**Figure 2:**
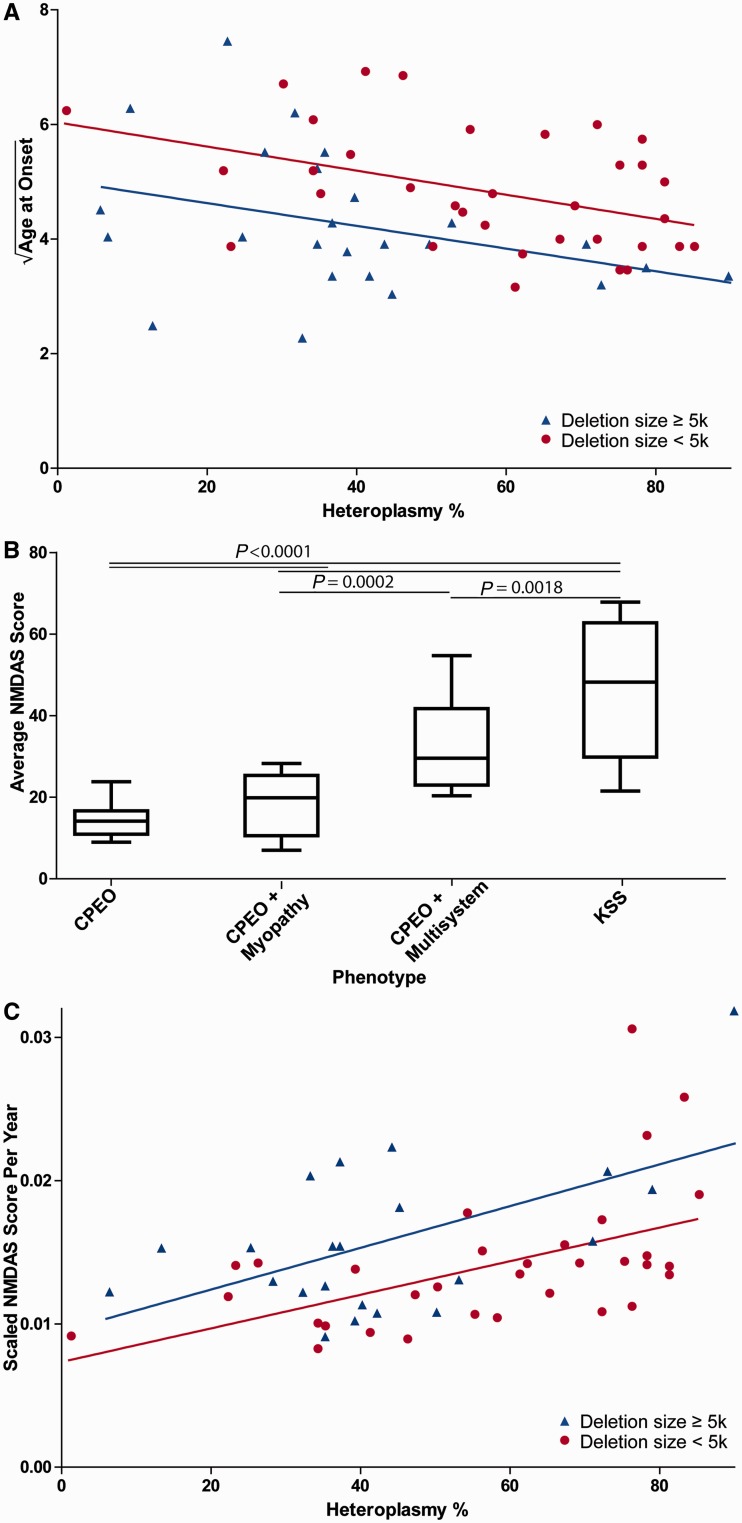
Heteroplasmy and deletion size are linearly correlated with age at onset and NMDAS score progression. (**A**) Age at onset is predicted by both mitochondrial DNA heteroplasmy and deletion size. The *y*-axis shows the square root of age at onset. Data are from our cohort (*n* = 60, R^2^ = 0.18). Both mitochondrial DNA heteroplasmy (*P* = 0.0027) and deletion size (*P* = 0.0039) are significantly correlated with age at onset in our cohort using multiple regression. *P*-values are for both predictors as continuous variables, mitochondrial DNA deletion size is dichotomous for visualization only. (**B**) Phenotype and average NMDAS score are highly significantly correlated (*P* < 0.0001). Individual comparison *P*-values are shown. (**C**) NMDAS progression (scaled NMDAS points per year) is highly significantly correlated with both mitochondrial DNA deletion size (*P* < 0.0001) and heteroplasmy (*P* < 0.0001) (*n* = 55, R^2^ = 0.49). The *y*-axis shows scaled NMDAS score per year. *P*-values are for both predictors as continuous variables, deletion size is dichotomous for visualization only. KSS = Kearns-Sayre syndrome.

The square root of age at onset was used in all analyses, which was identified by Box-Cox as the optimal transform. For the subjects in our patient cohort with known age at onset (*n* = 60), age at onset was significantly correlated with both mitochondrial DNA deletion size (b = −0.41, *P* = 0.0039) and muscle mitochondrial DNA heteroplasmy (b = −0.42, *P* = 0.0027) using multiple linear regression (R^2^ = 0.18) (Fig. 2A). Similarly, in the meta-analysis (*n* = 117), both mitochondrial DNA deletion size (b = −0.30, *P* = 0.0008) and muscle mitochondrial DNA heteroplasmy (b = −0.30, *P* = 0.0010) were significantly correlated with age at onset (R^2^ = 0.15).

In a phenotypic analysis, patients identified with Kearns-Sayre syndrome were directly compared to those with CPEO or CPEO + myopathy; intermediate phenotypes (in particular non-Kearns-Sayre syndrome, multisystem disease phenotype) were excluded from this analysis. For such patients from our cohort (*n* = 64) we found that both mitochondrial DNA heteroplasmy (b = −1.4, *P* = 0.0020) and mitochondrial DNA deletion size (b = −0.74, *P* = 0.0318) were significantly correlated with phenotype using multiple logistic regression. Similarly, in the meta-analysis (*n* = 192), both mitochondrial DNA heteroplasmy (b = −0.56, *P* < 0.0001) and mitochondrial DNA deletion size (b = −0.18, *P* = 0.0453) were significantly correlated with phenotype.

Average NMDAS score correlated with traditional phenotype classification (Fig. 2B). Box-Cox identified the fourth root of the NMDAS score (NMDAS^0.25^) as the optimal transformation for linear regression; this was divided by the age at assessment to measure NMDAS progression. In our cohort (*n* = 55), we found that both mitochondrial DNA deletion size (b = 0.49, *P* < 0.0001) and mitochondrial DNA heteroplasmy (b = 0.70, *P* < 0.0001) were significant predictors of NMDAS progression using multiple regression (R^2^ = 0.49) (Fig. 2C).

### Disease burden and progression of patients with a common mitochondrial DNA deletion are correlated with heteroplasmy

We also investigated the cohort of patients whose mitochondrial DNA disease is associated with a common 4977 bp single, large-scale mitochondrial DNA deletion (Schon *et al.*, 1989). Using a combined data set of the meta-analysis and our own patient cohort, we observed that muscle mitochondrial DNA heteroplasmy was a significant predictor of both clinical phenotype [*n* = 85, odds ratio for 10% change in heteroplasmy 1.43, 95% confidence interval (CI) 1.13–1.81, *P* = 0.0030] and also age of disease onset (*n* = 37, r = −0.44, *P* = 0.0063).

### COX-deficient fibre density is dependent on muscle heteroplasmy and deletion location but not deletion size

We next studied the relationship between the proportion of COX-deficient muscle fibres in patient biopsies, levels of muscle mitochondrial DNA heteroplasmy and *MT-CO* gene deletion (namely, deletion of part or all of at least one of the *MT-CO1*, *MT-CO2* or *MT-CO3* genes within the deleted mitochondrial DNA molecule). The square root of COX-deficient fibre density was used in all analyses, which was identified by Box-Cox as the optimal transform.

**Figure 3:**
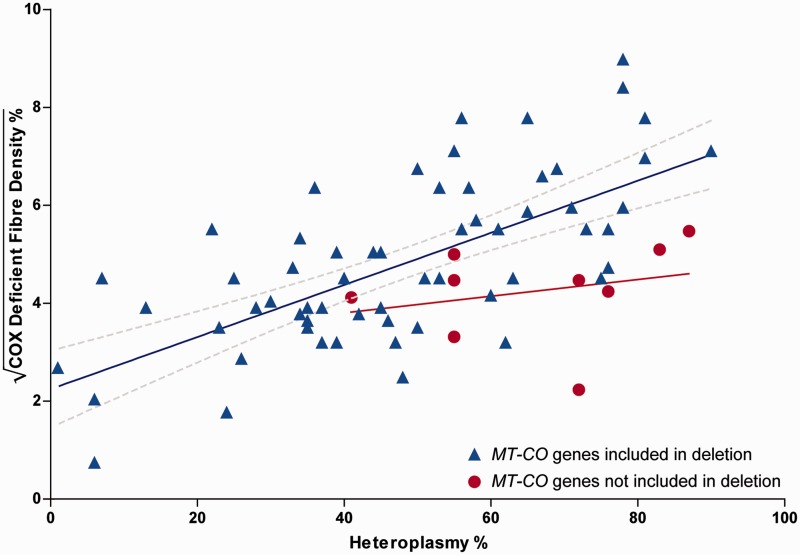
COX-deficient fibre density is dependent on skeletal muscle mitochondrial DNA heteroplasmy and deletion of *MT-CO* genes. The *y*-axis shows the square root of the COX-deficient fibre density %. Data are from our cohort, *n* = 72, R^2^ = 0.43. Heteroplasmy (*P* < 0.0001) and deletion of *MT-CO* genes (*P* = 0.0018) are both significant predictors. Separate regression lines are shown for those that delete one or more *MT-CO* genes (*n* = 63, 95% CI for regression line shown) and those that do not (*n* = 9, gradient of regression line is not significantly non-zero, CI not shown).

In our patient cohort (*n* = 72), we observed that both muscle mitochondrial DNA heteroplasmy (b = 0.68, *P* < 0.0001) and *MT-CO* gene deletion (b = 0.31, *P* = 0.0018) were significantly correlated with COX-deficient fibre density using multiple linear regression (R^2^ = 0.43) (Fig. 3). Similarly, in the meta-analysis (*n* = 39), both mitochondrial DNA heteroplasmy (b = 0.49, *P* = 0.0012) and *MT-CO* gene deletion (b = 0.34, *P* = 0.0192) were significantly correlated with COX-deficient fibre density (R^2^ = 0.31).

As the inclusion of *MT-CO* genes within the deleted mitochondrial DNA region was significantly correlated with a larger mitochondrial DNA deletion size in both our cohort and the meta-analysis, we also examined whether there was a correlation between mitochondrial DNA deletion size and COX-deficient fibre density. Using multiple regression with all three predictors, in our patient cohort (*n* = 72) mitochondrial DNA deletion size (b = −0.081, *P* = 0.5104) was not a significant predictor, although both mitochondrial DNA heteroplasmy (b = 0.65, *P* < 0.0001) and *MT-CO* gene deletion (b = 0.35, *P* = 0.0028) remained significant (R^2^ = 0.44). Similarly, in the meta-analysis (*n* = 39), mitochondrial DNA deletion size (b = −0.00010, *P* = 0.5586) was not a significant predictor; however, both mitochondrial DNA heteroplasmy (b = 0.48, *P* = 0.0016) and *MT-CO* gene deletion (b = 0.43, *P* = 0.0445) remained significant (R^2^ = 0.32).

### Longitudinal mixed modelling shows that mitochondrial DNA heteroplasmy, mitochondrial DNA deletion size and location are predictors of disease progression

**Figure 4:**
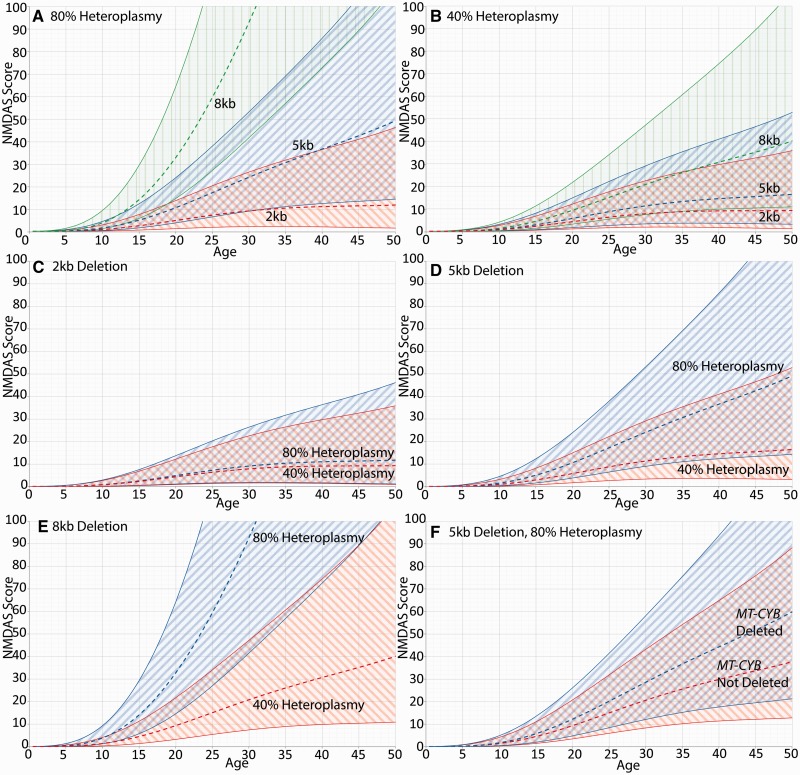
The effect of mitochondrial DNA deletion size and heteroplasmy on NMDAS progression. All panels show 95% CI. (**A** and **B**) The effect of mitochondrial DNA deletion size at 80% and 40% heteroplasmy, respectively. Deletion size is shown to have a greater impact at high heteroplasmy than at low heteroplasmy. (**C–E**) The effect of mitochondrial DNA heteroplasmy for a 2.0 kb, 5.0 kb and 8.0 kb mitochondrial DNA deletion, respectively. For small deletions the effect of heteroplasmy on NMDAS progression is negligible, but for larger deletions the effect is substantial. (**F**) The effect of deletion location for a 5.0 kb deletion present at 80% heteroplasmy; progression is faster when *MT-CYB* is included in the deleted region. **A–E** are generated from a model using time, deletion size and heteroplasmy as predictors. The model used for **F** has an additional deletion location predictor (*MT-CYB* gene inclusion).

Using longitudinal mixed modelling we showed that muscle mitochondrial DNA heteroplasmy (*P* < 0.0001) and mitochondrial DNA deletion size (*P* < 0.0001) were both highly significantly correlated with NMDAS progression in our patient cohort (*n* = 55) (Fig. 4). The interaction between mitochondrial DNA deletion size and mitochondrial DNA heteroplasmy was also significant (*P* = 0.0046), which is exemplified by comparing Fig. 4A with Fig. 4B, where mitochondrial DNA deletion size has a stronger effect at high mitochondrial DNA heteroplasmy levels than low mitochondrial DNA heteroplasmy levels; and Fig. 4C with Fig. 4E, where mitochondrial DNA heteroplasmy has a strong effect with large mitochondrial DNA deletions but negligible effect with small mitochondrial DNA deletions.

The location of the mitochondrial DNA deletion within the mitochondrial genome was also shown to affect disease progression. With mitochondrial DNA heteroplasmy and mitochondrial DNA deletion size in the model, deletion of the *MT-CYB* gene is significantly predictive of faster progression (*P* = 0.0085, *n* = 52) (Fig. 4F).

### Individual patient progression can be modelled longitudinally

As mixed modelling allows incorporation of random effects to model unknown variance, we were also able to use the clinical and molecular genetic data obtained to model the progress of individual patients and predict their expected course of disease development.

**Figure 5:**
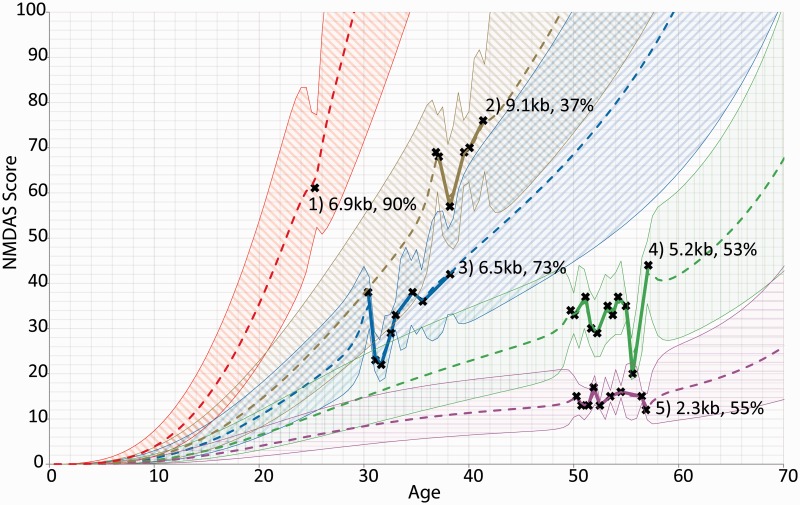
Longitudinal modelling of five individual patients with single, large-scale mitochondrial DNA deletion disease. The chosen patients are representative of the range of rates of disease progression found in our cohort. Actual NMDAS assessment scores are depicted as crosses joined by solid lines. Each patient is shown with their predicted progression trendline with 95% prediction intervals, and is labelled with deletion size and heteroplasmy. Only Patient 2 includes part of the *MT-CYB* gene in their deletion.

We studied five individual patients using a predictive model that incorporated muscle mitochondrial DNA heteroplasmy, mitochondrial DNA deletion size and involvement of *MT-CYB* gene deletion as predictive factors (Fig. 5). Expected progression is shown with 95% prediction intervals. The effect of mitochondrial DNA deletion size is exemplified by comparing Patients 4 and 5, as these only differ in this single parameter. The effect of mitochondrial DNA heteroplasmy is shown by comparing Patients 1 and 3, who have similarly sized mitochondrial DNA deletions (6.9 kb and 6.5 kb, respectively) but at differing mitochondrial DNA heteroplasmy levels. Patient 2 (9.1kb mitochondrial DNA deletion) shows a rapid progression despite a low level of mitochondrial DNA heteroplasmy (37% mitochondrial DNA deletion load), on account of the exceptionally large mitochondrial DNA deletion, whereas Patient 1 demonstrates that even a single NMDAS score can represent a useful prognostic input.

## Discussion

The aim of our study was to further understanding of the nature and progression of mitochondrial DNA disease. This remains challenging despite the fact that the first mitochondrial DNA mutations were described 25 years ago (Holt *et al.*, 1988). The relationship between a specific mitochondrial DNA mutation and clinical phenotype and progression is complex for many common mitochondrial DNA mutations not least because of heteroplasmy and variation in mitochondrial DNA mutation load. In addition, a particular mitochondrial DNA mutation can cause several different disease phenotypes depending on specific organ involvement and these can be difficult to categorize without the benefit of a scale of disease burden. This study has focused on patients with single, large-scale mitochondrial DNA deletions as these represent a common cause of mitochondrial disease and exemplify many of the challenges that are observed with other mitochondrial DNA defects. We have identified that muscle mitochondrial DNA heteroplasmy levels, mitochondrial DNA deletion size and mitochondrial DNA deletion location are all important in understanding the expression and progression of clinical disease.

Two major factors have helped to define the relationship between single, large-scale mitochondrial DNA deletions and disease progression. The first is the use of a validated rating scale that covers the major clinical features of mitochondrial disease. Traditional phenotyping of mitochondrial disease is difficult because of the frequent involvement of multiple systems. The NMDAS, as a quantitative measure of mitochondrial disease burden, allows us to study disease progression with unprecedented power, and longitudinal modelling of successive NMDAS assessments for patients demonstrates the benefits of such an approach. Second is the use of statistical techniques, including multiple regression analyses that we have applied to our own data and those previously published in the literature, using the Box-Cox transformation to identify optimal transformations to normality. In trying to understand the nature and progression of single, large-scale mitochondrial DNA deletion disease, where there is intercorrelation between the predictive factors, these techniques are required to correctly identify significant findings; indeed, the reanalysis of previously reported data using these techniques has clarified previous uncertainties.

Our longitudinal modelling shows that disease burden and progression is predicted by muscle mitochondrial DNA heteroplasmy level, mitochondrial DNA deletion size and the location of the mitochondrial DNA deletion within the mitochondrial genome. These findings are reinforced by a series of results, from both our cohort and the meta-analysis, demonstrating that both phenotype and age at onset are predicted by mitochondrial DNA deletion size and mitochondrial DNA heteroplasmy, and also by the significance of heteroplasmy as a predictor in a cohort of patients with the common 4977 bp mitochondrial DNA deletion. Previous studies have been contradictory regarding the use of these factors as predictors, reporting that both phenotype and age at onset were dependent on mitochondrial DNA deletion size, but not significantly related to mitochondrial DNA heteroplasmy (Yamashita *et al.*, 2008), or that the clinical phenotype was related to mitochondrial DNA heteroplasmy, but unrelated to mitochondrial DNA deletion size, although age at onset was related to both factors (López-Gallardo *et al.*, 2009). Skeletal muscle mitochondrial DNA heteroplasmy and mitochondrial DNA deletion size have also been reported as not being useful in disease progression prediction (Auré *et al.*, 2007). More seminal studies also reported inconsistently on the link between these predictors and phenotype or age at onset (Holt *et al.*, 1989; Moraes *et al.*, 1989; Goto *et al.*, 1990). However, we found that multiple regression identifies consistent correlation between disease phenotype or progression and both mitochondrial DNA heteroplasmy and deletion size. Furthermore, multiple regression of previously published data is revealing; for example, whereas López-Gallardo *et al.* (2009) found that mitochondrial DNA deletion size is not predictive of phenotype (*P* = 0.3953), if we perform logistic regression on their same data, but use multiple regression with deletion size and heteroplasmy, deletion size is indeed a significant predictor of phenotype (*P* = 0.0330); in this case, the negative correlation between heteroplasmy and deletion size leads to the masking of the effect of deletion size when simple linear regression is used.

The correlations between predictive factors are of interest in themselves. A negative correlation between muscle heteroplasmy levels and mitochondrial DNA deletion size was noted by López-Gallardo *et al.* (2009) who surmized from this that shorter mitochondrial DNA deletions do not have a replicative advantage. We would speculate that the observed correlation between muscle mitochondrial DNA heteroplasmy and mitochondrial DNA deletion size most likely reflects the spectrum of disease that presents with a clinically recognizable phenotype within the population, rather than any intrinsic biochemically, or otherwise, driven relationship. Thus, individuals with small mitochondrial DNA deletions present at low heteroplasmy levels would not present clinically with symptoms, which is consistent with the observation that potentially pathogenic mitochondrial DNA mutations are widespread in the non-diseased population but at sub-threshold levels (Elliott *et al.*, 2008).

We have found that deletions including the *MT-CYB* gene are related to faster progression, which is consistent with the report by López-Gallardo *et al.* (2009) that deletion of the *MT-CYB* gene was linked to a more severe phenotype. However, the reason for this remains uncertain, but could potentially indicate that complex III deficiency has a particularly important role in the disease mechanism.

We also identified that *MT-CO* deletion, that is involvement of either *MT-CO1*, *MT-CO2* or *MT-CO3* in the deleted region, is predictive of COX-deficient fibre density. Though previous studies reported no such link (Goto *et al.*, 1990; Oldfors *et al.*, 1992), re-analysis of these studies using multiple regression reveals the same trend in all cases, and at statistical significance when the study is large enough (Goto *et al.*, 1990). The impact of mitochondrial DNA deletion location on COX-deficient fibre density, and the consistent correlation between mitochondrial DNA deletion size and disease burden and progression, brings into question the hypothesis that mitochondrial–transfer RNA genes are the root of the pathogenicity of mitochondrial DNA deletions (Schon *et al.*, 2012), which is predicated on the lack of correlation between the site of the mitochondrial DNA deletion and the pathogenicity. However, a more comprehensive data set would be required to elucidate the relative importance of mitochondrial–transfer RNA genes, specific oxidative phosphorylation protein genes, and other potential pathogenic mechanisms as regards biochemical defects and the resulting clinical phenotype.

An important implication of this study regards the threshold level for pathogenicity of a mitochondrial DNA deletion (Rossignol *et al.*, 2003). The co-dependence of disease burden on mitochondrial DNA heteroplasmy and mitochondrial DNA deletion size and location, and in particular the interaction of these quantities, implies that the threshold for phenotypic expression is likely to be dependent on the size and location of the deletion.

Although our studies provide new insights into disease progression in patients with single, large-scale mitochondrial DNA deletions, further work is still required. First, it should be considered that both mitochondrial DNA deletion size and our location parameter are perhaps merely proxies for the underlying pathogenic nature of the mitochondrial DNA deletion; a more nuanced characterization of these mitochondrial DNA deletions may better predict pathogenesis and mitochondrial disease progression. However, such a model would potentially involve a large number of parameters, in which case a larger data set would be required to achieve reasonable statistical power. Second, it is notable that we do not have any predictor that encapsulates the sometimes multi-system nature of disease. In this regard, Auré *et al.* (2007) found that the presence of the mutation in blood was predictive of a neurological phenotype, and a similar trend in urine, a result confirmed by Blackwood *et al.* (2010) with regard to severe early onset disease as compared to milder phenotypic presentation. These reports suggest that blood or urine mitochondrial DNA heteroplasmy levels, together with skeletal muscle mitochondrial DNA heteroplasmy, may lead to an improved prediction of disease prognosis. Third, in this study we have only considered progression of the overall disease burden; modelling of progression in individually affected systems should provide useful understanding to clinicians with regard to patient care.

There are additional limitations to be acknowledged. First, though most of the findings from our cohort are corroborated by the meta-analysis, there are differences in the two data sets; in particular the correlations between predictors are generally stronger in our cohort than the meta-analysis. These differences may arise in part from the fact that our data are more homogeneous, but also perhaps because the make-up of our cohort is different; we have relatively few patients presenting with a Kearns-Sayre syndrome phenotype compared with the meta-analysis. Secondly, our studies, and those selected for the meta-analysis have concentrated on muscle heteroplasmy levels. Finally, though we do not employ Bonferroni adjustment in most of our analyses for reasons well documented in the literature and particularly as we are testing *a priori* hypotheses (Thomas, 1998), the large number of statistical tests used do open up the potential for more frequent type I statistical errors. However, consistent findings in both our cohort and the meta-analysis provide support for firm conclusions to be drawn.

Our finding that deletion size, heteroplasmy, and deletion location are predictors of disease progression is important for all clinicians looking after patients with single, large-scale mitochondrial DNA deletions. We have therefore developed a web-based tool (http://research.ncl.ac.uk/mitoresearch) to support clinicians in their management of these patients.

In conclusion, we have demonstrated that skeletal muscle mitochondrial DNA heteroplasmy, mitochondrial DNA deletion size and deletion location are predictive of disease severity and progression, and that applied with NMDAS, a quantitative measure of total disease burden, we are able to longitudinally model disease progression both at a population level and for individual patients. This means that advice and care plans for patients can be given on an individual basis. In addition, understanding the natural history of disease is crucial if we are to assess the benefits of therapeutic treatments. Thus we strongly recommend that all patients with single, large scale mitochondrial DNA deletion disease have both a detailed analysis of the muscle biopsy and clinical evaluation using the NMDAS rating scale.

## Supplementary Material

Supplementary Data

## Abbreviations

COX: cytochrome *c* oxidase
CPEO: chronic progressive external ophthalmoplegia
NMDAS: Newcastle Mitochondrial Disease Adult Scale
