# Supplementary material for: Disease progression in patients with single, large-scale mitochondrial DNA deletions
Source: Brain. 2013 Nov 23;137(2):323–34. doi: 10.1093/brain/awt321 (PMC3914470; doi:10.1093/brain/awt321)
Supplement: Supplementary Data [file supp_137_2_323__index.html]

Disease progression in patients with single, large-scale mitochondrial DNA deletions — Disease progression in patients with single, large-scale mitochondrial DNA deletions — Supplementary Data 

# Disease progression in patients with single, large-scale mitochondrial DNA deletions

## Supplementary Data

files

**Files in this Data Supplement:**

- Supplementary Data - doc file
