# Supplementary material for: Disease progression in patients with single, large-scale mitochondrial DNA deletions
Source: Brain. 2013 Nov 23;137(2):323–34. doi: 10.1093/brain/awt321 (PMC3914470; doi:10.1093/brain/awt321)
Supplement: Supplementary Data [file supp_awt321_brain-2013-01156-File009.doc]

# Supplementary Tables

Supplementary Table 1 Data from our cohort.

| **No** | **Phenotype** | **Age at**  **Onset** | **Age at**  **Biopsy** | **COX**  **%** | **Deletion**  **Size** | **Break**  **point 5’** | **Break**  **Point 3'** | **No. NMDAS**  **Assessments** | **Het**  **%** |
| --- | --- | --- | --- | --- | --- | --- | --- | --- | --- |
| 1 | KSS | 11 | 27 | 10 | 9120 | 6468 | 15588 | 7 | 37% |
| 2 | Multisystem | 6 | 46 | 15 | 8704 | 7175 | 15879 | 4 | 13% |
| 3 | CPEO |  | 57 | 4 | 8560 | 5942 | 14502 |  | 6% |
| 4 | CPEO | 30 | 34 | 40 | 8039 | 7637 | 15676 | 3 | 36% |
| 5 | CPEO |  | 65 | 25 | 7977 | 6537 | 14514 |  | 45% |
| 6 | CPEO | 38 |  |  | 7958 | 6033 | 13991 | 1 | 32% |
| 7 | CPEO + MM | 15 | 25 | 25 | 7768 | 6352 | 14120 | 7 | 44% |
| 8 | Multisystem | 14 | 59 | 10 | 7676 | 6323 | 13999 | 5 | 39% |
| 9 | Multisystem | 55 | 85 | 12 | 7671 | 6741 | 14412 |  | 23% |
| 10 | Multisystem | 5 | 28 | 22 | 7648 | 6341 | 13989 | 4 | 33% |
| 11 | CPEO | 20 | 38 | 0.5 | 7595 | 7845 | 15440 | 4 | 6% |
| 12 | CPEO | 39 | 72 | 5 | 7500 |  |  |  | 10% |
| 13 | CPEO | 30 |  | 15 | 7498 | 7130 | 14628 | 2 | 28% |
| 14 |  |  | 113 |  | 7451 | 8287 | 15738 |  | 5% |
| 15 | CPEO + MM |  | 60 | 3 | 7355 | 7168 | 14523 |  | 24% |
| 16 | CPEO + MM |  | 59 | 20 | 7284 | 6774 | 14058 |  | 51% |
| 17 | CPEO | 18 | 30 | 15 | 7144 | 5772 | 12916 | 4 | 37% |
| 18 | CPEO | 16 | 16 | 20 | 7129 | 8543 | 15672 |  | 7% |
| 19 | CPEO + MM |  | 68 |  | 6978 | 7821 | 14799 |  | 56% |
| 20 | CPEO | 27 | 63 | 13 | 6864 | 7128 | 13992 | 3 | 35% |
| 21 | KSS | 11 | 23 | 50 | 6864 | 7128 | 13992 | 1 | 90% |
| 22 | CPEO + MM | 22 | 36 | 20 | 6549 | 6006 | 12555 | 6 | 40% |
| 23 | KSS | 10 | 24 | 30 | 6472 | 7540 | 14012 | 8 | 73% |
| 24 | Multisystem | 15 | 41 | 45 | 6058 | 8838 | 14896 | 2 | 50% |
| 25 | CPEO + MM |  |  | 15 | 5958 | 6002 | 11960 |  | 45% |
| 26 | CPEO |  | 41 |  | 5906 | 8324 | 14230 |  | 20% |
| 27 | CPEO | 15 | 40 | 12 | 5899 | 9523 | 15422 | 7 | 35% |
| 28 | CPEO | 16 | 33 | 20 | 5813 | 9754 | 15567 | 1 | 25% |
| 29 | CPEO + MM | 15 | 34 | 35 | 5470 | 6603 | 12073 | 7 | 71% |
| 30 | Multisystem | 9 | 31 | 25 | 5340 | 6714 | 12054 | 5 | 45% |
| 31 | Multisystem | 18 | 49 | 20 | 5160 | 9258 | 14418 | 11 | 53% |
| 32 | Multisystem | 12 | 32 | 49 | 5000 |  |  | 4 | 79% |
| 33 | Multisystem | 11 | 56 | 16 | 5000 |  |  | 1 | 42% |
| 34 | Multisystem | 12 | 20 | 20 | 4999 | 6625 | 11624 |  | 75% |
| 35 | CPEO | 23 | 51 | 32 | 4977 | 8470 | 13447 | 8 | 58% |
| 36 | Multisystem | 28 | 40 | 20 | 4977 | 8470 | 13447 | 7 | 75% |
| 37 | CPEO + MM | 14 | 31 | 10 | 4977 | 8470 | 13447 | 6 | 62% |
| 38 | Multisystem | 16 | 37 | 43 | 4977 | 8470 | 13447 | 6 | 67% |
| 39 | Multisystem | 10 | 34 | 30 | 4977 | 8470 | 13447 | 5 | 61% |
| 40 | CPEO + MM | 27 | 55 | 14 | 4977 | 8470 | 13447 | 5 | 34% |
| 41 | Multisystem | 28 | 41 | 80 | 4977 | 8470 | 13447 | 5 | 78% |
| 42 | CPEO | 20 | 28 |  | 4977 | 8470 | 13447 | 3 | 54% |
| 43 | KSS | 12 | 15 | 30 | 4977 | 8470 | 13447 | 2 | 76% |
| 44 | KSS | 15 | 33 | 35 | 4977 | 8470 | 13447 | 1 | 78% |
| 45 | CPEO + MM | 19 | 43 | 60 | 4977 | 8470 | 13447 | 1 | 81% |
| 46 | CPEO + MM | 33 | 41 | 70 | 4977 | 8470 | 13447 | 1 | 78% |
| 47 | CPEO |  | 35 | 8 | 4977 | 8470 | 13447 | 1 | 26% |
| 48 | CPEO + MM |  | 43 | 30 | 4977 | 8470 | 13447 | 1 | 56% |
| 49 | CPEO + MM | 18 | 57 | 40 | 4977 | 8470 | 13447 |  | 57% |
| 50 | CPEO | 45 |  | 16 | 4977 | 8470 | 13447 |  | 30% |
| 51 | CPEO |  | 18 | 6 | 4977 | 8470 | 13447 |  | 48% |
| 52 | CPEO + MM |  | 25 |  | 4977 | 8470 | 13447 |  | 71% |
| 53 |  |  | 37 | 14 | 4977 | 8470 | 13447 |  | 42% |
| 54 | CPEO |  | 39 | 60 | 4977 | 8470 | 13447 |  | 65% |
| 55 | CPEO + MM |  | 40 | 20 | 4977 | 8470 | 13447 |  | 63% |
| 56 | CPEO |  | 46 |  | 4977 | 8470 | 13447 |  | 13% |
| 57 | CPEO + MM | 15 |  |  | 4963 | 10105 | 15068 | 1 | 23% |
| 58 | KSS |  | 16 |  | 4959 | 8474 | 13433 |  | 88% |
| 59 | CPEO | 39 | 61 | 7 | 4909 | 8814 | 13723 | 4 | 1% |
| 60 | CPEO | 37 |  | 28 | 4885 | 7205 | 12090 | 1 | 34% |
| 61 | CPEO |  | 27 |  | 4851 | 10747 | 15598 |  | 65% |
| 62 | CPEO | 23 | 63 | 15 | 4770 | 9349 | 14119 | 1 | 35% |
| 63 | CPEO + MM |  |  | 60 | 4770 | 9349 | 14119 |  | 56% |
| 64 | CPEO | 15 | 40 | 12 | 4752 | 8289 | 13041 | 9 | 50% |
| 65 | Multisystem | 15 | 26 | 26 | 4641 | 10946 | 15587 | 3 | 83% |
| 66 | CPEO + MM | 34 | 38 | 34 | 4604 | 9057 | 13661 | 5 | 65% |
| 67 | KSS |  | 17 | 17 | 4599 | 9752 | 14351 |  | 60% |
| 68 | CPEO |  | 41 | 15 | 4596 | 9528 | 14124 |  | 35% |
| 69 | KSS | 15 | 29 | 50 | 4500 |  |  | 9 | 85% |
| 70 | Multisystem | 27 | 58 | 30 | 4392 | 8576 | 12968 | 4 | 22% |
| 71 | CPEO + MM | 21 | 32 | 45 | 4382 | 8586 | 12968 | 6 | 69% |
| 72 | CPEO | 21 | 25 | 40 | 4372 | 8929 | 13301 |  | 53% |
| 73 | Multisystem | 30 | 45 | 25 | 4241 | 9498 | 13739 | 8 | 39% |
| 74 | Multisystem | 25 | 40 | 48 | 4237 | 9486 | 13723 | 6 | 81% |
| 75 | CPEO | 47 | 60 | 13 | 4237 | 9486 | 13723 | 6 | 46% |
| 76 | CPEO |  | 70 | 22 | 4223 | 9500 | 13723 |  | 76% |
| 77 | CPEO + MM | 16 | 32 | 20 | 4113 | 11262 | 15375 | 9 | 72% |
| 78 | KSS | 15 |  |  | 3979 | 11657 | 15636 |  | 50% |
| 79 | Multisystem | 24 | 44 | 10 | 3693 | 9756 | 13449 | 7 | 47% |
| 80 | CPEO |  | 63 | 50 | 3527 | 7729 | 11256 |  | 55% |
| 81 |  |  | 105 | 11 | 3039 | 10950 | 13989 |  | 55% |
| 82 | Multisystem | 36 | 50 | 5 | 2803 | 11637 | 14440 | 11 | 72% |
| 83 | CPEO + MM |  | 40 | 30 | 2308 | 12113 | 14421 |  | 87% |
| 84 | CPEO | 35 | 50 | 20 | 2300 | 12112 | 14412 | 9 | 55% |
| 85 | Multisystem | 48 | 72 | 17 | 2300 | 12112 | 14412 | 9 | 41% |
| 86 | CPEO |  |  | 18 | 2300 | 12112 | 14412 | 1 | 76% |
| 87 | CPEO |  | 56 | 25 | 2297 | 12115 | 14412 |  | 55% |

Supplementary Table 2 Data used in the meta-analysis.

| **No** | **Original**  **Study** | **Phenotype** | **Age at**  **Onset** | **Age at**  **Biopsy** | **COX**  **%** | **Del**  **Size** | **Break**  **point 5’** | **Break**  **point 3’** | **Het%** | **In López-Gallardo *et al.*, 2009** |
| --- | --- | --- | --- | --- | --- | --- | --- | --- | --- | --- |
| 1 | López-Gallardo *et al.*, 2009 | KSS |  |  |  | 11041 | 4166 | 15207 | 84% | Yes |
| 2 | Marin-Garcia *et al.*, 2002 | KSS | 3 | 15 |  | 11000 | 4500 | 15500 | 20% | Yes |
| 3 | Kornblum *et al.*, 2005b | CPEO | 55 | 59 |  | 10900 |  |  | 23% | Yes |
| 4 | Kornblum *et al.*, 2005b | CPEO | 56 | 60 |  | 10900 |  |  | 49% | Yes |
| 5 | De Coo *et al.*, 1997 |  |  |  |  | 10000 |  |  | 1% | No |
| 6 | Solano *et al.*, 2003 | KSS | 8 | 17 |  | 9438 | 6003 | 15441 | 87% | Yes |
| 7 | Gellerich 2002 |  |  | 0 | 27 | 9000 | 6000 | 15000 | 69% | No |
| 8 | Fromenty *et al.*, 1996 | CPEO | 3 | 18 |  | 8800 |  |  | 32% | Yes |
| 9 | Ishikawa 2000 |  |  |  |  | 8731 | 6903 | 15634 | 55% | No |
| 10 | Emma *et al.*, 2006 | KSS | 1 | 14 |  | 8661 | 7836 | 16497 | 60% | Yes |
| 11 | López-Gallardo *et al.*, 2009 | KSS | 6 |  |  | 8477 | 6123 | 14600 | 82% | Yes |
| 12 | Solano *et al.*, 2003 | KSS | 9 | 20 |  | 8431 | 7515 | 15946 | 86% | Yes |
| 13 | Degoul *et al.*, 1991 | CPEO | 28 | 45 |  | 8137 | 5786 | 13923 | 25% | Yes |
| 14 | Simaan *et al.*, 1999 | KSS | 3 | 13 |  | 8000 |  |  | 18% | Yes |
| 15 | De Coo *et al.*, 1997 | KSS |  |  |  | 8000 |  |  | 60% | Yes |
| 16 | Blok *et al.*, 1995 | KSS | 10 | 14 |  | 7865 | 6238 | 14103 | 25% | Yes |
| 17 | Heddi *et al.*, 1994 | KSS |  |  |  | 7768 | 7669 | 15437 | 74% | Yes |
| 18 | Goto *et al.*, 1990 | CPEO | 7 | 15 | 14.5 | 7700 | 6000 | 13700 | 20% | Yes |
| 19 | Matsuoka *et al.*, 1992 | CPEO Multisystem |  | 15 |  | 7700 |  |  | 20% | Yes |
| 20 | Matsuoka *et al.*, 1992 | KSS |  | 21 |  | 7700 |  |  | 20% | Yes |
| 21 | Goto *et al.*, 1990 | CPEO Multisystem | 7 | 7 | 15.3 | 7700 | 6000 | 13700 | 30% | Yes |
| 22 | Goto *et al.*, 1990 | CPEO | 9 | 16 | 27.2 | 7700 | 6000 | 13700 | 40% | Yes |
| 23 | Matsuoka *et al.*, 1992 | CPEO |  | 16 |  | 7700 |  |  | 40% | Yes |
| 24 | Sadikovic 2010 | Multisystem |  | 40 |  | 7673 | 6331 | 14004 | 24% | No |
| 25 | Solano *et al.*, 2003 | KSS |  |  |  | 7663 | 6331 | 13994 | 64% | Yes |
| 26 | Johns *et al.*, 1989 | CPEO |  |  |  | 7650 | 6250 | 13900 | 25% | Yes |
| 27 | Montiel-Sosa, 2013 |  |  | 10 |  | 7628 | 7437 | 15065 | 85% | No |
| 28 | Sadikovic 2010 | CPEO |  | 59 |  | 7603 | 8469 | 16072 | 22% | No |
| 29 | Kiyomoto *et al.*, 1997 | CPEO Multisystem | 6 | 12 | 15 | 7565 | 7827 | 15392 | 66% | Yes |
| 30 | Sadikovic 2010 | CPEO + MM |  | 48 |  | 7544 | 7865 | 15409 | 14% | No |
| 31 | Oldfors 1992 |  |  | 56 | 9 | 7534 | 8366 | 15900 | 52% | No |
| 32 | Oldfors 1992 |  |  | 5 | 30 | 7534 | 8366 | 15900 | 73% | No |
| 33 | Odoardi *et al.*, 2003 | KSS | 7 | 21 |  | 7521 | 7983 | 15504 | 38% | No |
| 34 | Schroder *et al.*, 2000 | CPEO |  | 30 |  | 7500 |  |  | 15% | Yes |
| 35 | Kornblum *et al.*, 2005a | CPEO Multisystem | 10 | 34 |  | 7500 |  |  | 16% | Yes |
| 36 | Barrientos *et al.*, 1995 | CPEO Multisystem | 10 | 47 |  | 7500 |  |  | 23% | Yes |
| 37 | Kornblum *et al.*, 2005a | CPEO Multisystem | 14 | 42 |  | 7500 |  |  | 26% | Yes |
| 38 | Schroder *et al.*, 2000 | CPEO |  | 34 |  | 7500 |  |  | 26% | Yes |
| 39 | Schroder *et al.*, 2000 | CPEO |  | 26 |  | 7500 |  |  | 27% | Yes |
| 40 | Barrientos *et al.*, 1995 | KSS | 12 | 20 |  | 7500 |  |  | 40% | Yes |
| 41 | Odoardi *et al.*, 2003 | CPEO + MM | 8 | 18 |  | 7500 |  |  | 42% | No |
| 42 | Barrientos *et al.*, 1995 | CPEO Multisystem | 3 | 12 |  | 7500 |  |  | 50% | Yes |
| 43 | Kornblum *et al.*, 2005a | CPEO Multisystem | 10 | 38 |  | 7500 |  |  | 58% | Yes |
| 44 | Sadikovic 2010 | Multisystem |  | 18 |  | 7436 | 8637 | 16073 | 45% | No |
| 45 | Goto *et al.*, 1990 | CPEO | 13 | 31 | 17.2 | 7300 | 6000 | 13300 | 40% | Yes |
| 46 | Oldfors 1992 |  |  | 13 | 5 | 7300 | 8600 | 15900 | 43% | No |
| 47 | Sadikovic 2010 | Multisystem |  | 46 |  | 7213 | 8427 | 15640 | 47% | No |
| 48 | Sadikovic 2010 | KSS |  | 31 |  | 7213 | 8427 | 15640 | 82% | No |
| 49 | Matsuoka *et al.*, 1992 | CPEO |  | 16 |  | 7200 |  |  | 5% | Yes |
| 50 | Moraes *et al.*, 1992 | KSS |  |  |  | 7100 |  |  | 70% | Yes |
| 51 | Sadikovic 2010 | Multisystem |  | 6 |  | 7039 | 8623 | 15662 | 70% | No |
| 52 | Zeviani *et al.*, 1988 | KSS | 8 | 15 |  | 7025 | 5275 | 12300 | 45% | Yes |
| 53 | Schroder *et al.*, 2000 | KSS |  | 19 |  | 7000 |  |  | 20% | Yes |
| 54 | Kornblum *et al.*, 2005a | KSS | 12 | 31 |  | 7000 |  |  | 66% | Yes |
| 55 | Goto *et al.*, 1990 | CPEO | 22 | 49 | 11.6 | 6800 | 8600 | 15400 | 30% | Yes |
| 56 | López-Gallardo *et al.*, 2009 | CPEO | 31 |  |  | 6798 | 6024 | 12822 | 30% | Yes |
| 57 | Barrientos *et al.*, 1995 | KSS | 14 | 24 |  | 6500 |  |  | 56% | Yes |
| 58 | Kornblum *et al.*, 2005b | CPEO | 25 | 51 |  | 6500 |  |  | 58% | Yes |
| 59 | Schroder *et al.*, 2000 | CPEO |  | 50 |  | 6500 |  |  | 60% | Yes |
| 60 | Schroder *et al.*, 2000 | KSS |  | 21 |  | 6500 |  |  | 66% | Yes |
| 61 | Fromenty *et al.*, 1996 | KSS | 8 | 22 |  | 6495 | 7836 | 14331 | 31% | Yes |
| 62 | Solano *et al.*, 2003 | KSS | 12 | 13 |  | 6366 | 7949 | 14315 | 69% | Yes |
| 63 | López-Gallardo *et al.*, 2009 | CPEO |  |  |  | 6279 | 7409 | 13688 | 21% | Yes |
| 64 | Solano *et al.*, 2003 | CPEO |  |  |  | 6213 | 7407 | 13620 | 7% | Yes |
| 65 | Sadikovic 2010 | CPEO + MM |  | 26 |  | 6119 | 9516 | 15635 | 32% | No |
| 66 | Larsson and Holme, 1992 | KSS | 7 | 16 | 5 | 6100 | 8800 | 14900 | 59% | No |
| 67 | Kornblum *et al.*, 2005a | CPEO Multisystem | 8 | 61 |  | 6000 |  |  | 46% | Yes |
| 68 | Schroder *et al.*, 2000 | CPEO |  | 49 |  | 6000 |  |  | 46% | Yes |
| 69 | De Coo *et al.*, 1997 | CPEO |  |  |  | 6000 | 9500 | 15500 | 70% | Yes |
| 70 | De Coo *et al.*, 1997 |  |  |  |  | 6000 |  |  | 82% | No |
| 71 | Zeviani *et al.*, 1988 | KSS | 4 | 7 |  | 5980 | 9020 | 15000 | 66% | Yes |
| 72 | Solano *et al.*, 2003 | CPEO |  |  |  | 5928 | 9816 | 15744 | 29% | Yes |
| 73 | Sadikovic 2010 | KSS |  | 42 |  | 5905 | 8467 | 14372 | 70% | No |
| 74 | Marie *et al.*, 1999 | KSS | 1 | 5 |  | 5900 |  |  | 30% | Yes |
| 75 | Sadikovic 2010 | KSS |  | 11 |  | 5867 | 8558 | 14425 | 65% | No |
| 76 | Okulla *et al.*, 2005 | CPEO | 12 | 13 |  | 5800 |  |  | 32% | Yes |
| 77 | Sadikovic 2010 | CPEO + MM |  | 38 |  | 5630 | 8429 | 14059 | 33% | No |
| 78 | Kornblum *et al.*, 2005a | CPEO Multisystem | 28 | 52 |  | 5600 |  |  | 85% | Yes |
| 79 | Sudoyo *et al.*, 1993 | CPEO | 12 | 34 |  | 5500 | 8000 | 13500 | 39% | Yes |
| 80 | Kornblum *et al.*, 2005b | KSS | 16 | 47 |  | 5500 |  |  | 58% | Yes |
| 81 | Schroder *et al.*, 2000 | KSS |  | 40 |  | 5500 |  |  | 58% | Yes |
| 82 | Goto *et al.*, 1990 | KSS | 11 | 12 |  | 5500 | 7500 | 13000 | 80% | Yes |
| 83 | Zeviani *et al.*, 1988 | KSS | 3 | 12 |  | 5448 | 10600 | 16048 | 57% | Yes |
| 84 | Sadikovic 2010 | MM |  | 26 |  | 5438 | 8140 | 13578 | 11% | No |
| 85 | Goto *et al.*, 1990 | KSS | 2 | 13 | 23.9 | 5400 | 7000 | 12400 | 60% | Yes |
| 86 | Shanske *et al.*, 2002 | CPEO |  |  |  | 5355 | 10004 | 15359 | 40% | No |
| 87 | López-Gallardo *et al.*, 2009 | KSS |  |  |  | 5311 | 7450 | 12761 | 73% | Yes |
| 88 | Sadikovic 2010 | CPEO + MM |  | 57 |  | 5225 | 6076 | 11301 | 13% | No |
| 89 | Goto *et al.*, 1990 | KSS | 4 | 14 | 40.6 | 5200 | 8500 | 13700 | 90% | Yes |
| 90 | López-Gallardo *et al.*, 2009 | CPEO |  |  |  | 5113 | 8477 | 13590 | 80% | Yes |
| 91 | Sadikovic 2010 | Multisystem |  | 34 |  | 5112 | 8468 | 13580 | 50% | No |
| 92 | Goto *et al.*, 1990 | KSS | 12 | 30 | 14 | 5100 | 10059 | 15159 | 70% | Yes |
| 93 | Carta *et al.*, 2000 | CPEO |  | 28 |  | 5049 | 9570 | 14619 | 55% | Yes |
| 94 | Vazquez-Acevedo *et al.*, 1995 | KSS | 4 | 17 |  | 5026 | 10050 | 15076 | 86% | Yes |
| 95 | Johns and Hurko, 1989 | KSS | 13 | 43 |  | 5014 | 8708 | 13722 | 65% | Yes |
| 96 | Kornblum *et al.*, 2005a | CPEO Multisystem | 55 | 61 |  | 5000 |  |  | 5% | Yes |
| 97 | Schroder *et al.*, 2000 | CPEO |  | 39 |  | 5000 |  |  | 26% | Yes |
| 98 | Moraes *et al.*, 1992 | KSS |  |  |  | 5000 |  |  | 36% | Yes |
| 99 | Barrientos *et al.*, 1995 | CPEO Multisystem | 8 | 16 |  | 5000 |  |  | 42% | Yes |
| 100 | Kornblum *et al.*, 2005a | CPEO Multisystem | 12 | 50 |  | 5000 |  |  | 53% | Yes |
| 101 | Tanaka *et al.*, 1989 | CPEO | 14 | 32 |  | 5000 | 8600 | 13600 | 53% | Yes |
| 102 | Schroder *et al.*, 2000 | CPEO |  | 38 |  | 5000 |  |  | 53% | Yes |
| 103 | Schroder *et al.*, 2000 | KSS |  | 18 |  | 5000 |  |  | 54% | Yes |
| 104 | Moraes *et al.*, 1992 | CPEO |  |  |  | 5000 |  |  | 55% | Yes |
| 105 | Schroder *et al.*, 2000 | CPEO |  | 30 |  | 5000 |  |  | 55% | Yes |
| 106 | Schroder *et al.*, 2000 | KSS |  | 19 |  | 5000 |  |  | 58% | Yes |
| 107 | Vielhaber *et al.*, 2002 | CPEO |  | 44 |  | 5000 |  |  | 58% | Yes |
| 108 | Vielhaber *et al.*, 2002 | CPEO |  | 29 |  | 5000 |  |  | 68% | Yes |
| 109 | Matsuoka *et al.*, 1992 | CPEO |  | 14 |  | 5000 |  |  | 70% | Yes |
| 110 | Kornblum *et al.*, 2005b | CPEO | 30 | 48 |  | 5000 |  |  | 72% | Yes |
| 111 | Schroder *et al.*, 2000 | CPEO |  | 42 |  | 5000 |  |  | 72% | Yes |
| 112 | Vielhaber *et al.*, 2002 | CPEO |  | 47 |  | 5000 |  |  | 72% | Yes |
| 113 | Kornblum *et al.*, 2005a | CPEO Multisystem | 16 | 57 |  | 5000 |  |  | 74% | Yes |
| 114 | Vielhaber *et al.*, 2002 | KSS |  | 13 |  | 5000 |  |  | 74% | Yes |
| 115 | Moraes *et al.*, 1992 | KSS |  |  |  | 5000 |  |  | 76% | Yes |
| 116 | Kornblum *et al.*, 2005a | KSS | 9 | 39 |  | 5000 |  |  | 78% | Yes |
| 117 | Schroder *et al.*, 2000 | KSS |  | 29 |  | 5000 |  |  | 78% | Yes |
| 118 | Sadikovic 2010 | PEO |  | 24 |  | 4995 | 5835 | 10830 | 58% | No |
| 119 | López-Gallardo *et al.*, 2009 | CPEO |  |  |  | 4978 | 8482 | 13460 | 6% | Yes |
| 120 | Wong, 2001 | KSS |  | 26 |  | 4978 | 8482 | 13460 | 10% | Yes |
| 121 | Odoardi *et al.*, 2003 | CPEO | 39 | 43 |  | 4978 | 8482 | 13460 | 12% | Yes |
| 122 | Bernes *et al.*, 1993 | CPEO | 16 | 24 |  | 4978 | 8482 | 13460 | 15% | Yes |
| 123 | Pineda *et al.*, 2004 | CPEO |  |  |  | 4978 | 8482 | 13460 | 15% | Yes |
| 124 | López-Gallardo *et al.*, 2009 | CPEO |  |  |  | 4978 | 8482 | 13460 | 16% | Yes |
| 125 | Goto *et al.*, 1990 | CPEO | 20 | 53 | 5 | 4978 | 8482 | 13460 | 20% | Yes |
| 126 | Odoardi *et al.*, 2003 | CPEO | 12 | 14 |  | 4978 | 8482 | 13460 | 26% | Yes |
| 127 | Odoardi *et al.*, 2003 | CPEO | 35 | 45 |  | 4978 | 8482 | 13460 | 31% | Yes |
| 128 | Odoardi *et al.*, 2003 | CPEO | 15 | 36 |  | 4978 | 8482 | 13460 | 33% | Yes |
| 129 | Wong, 2001 | KSS |  | 28 |  | 4978 | 8482 | 13460 | 33% | Yes |
| 130 | Degoul *et al.*, 1991 | CPEO Multisystem | 4 | 31 |  | 4978 | 8482 | 13460 | 34% | Yes |
| 131 | Gellerich 2002 |  |  |  |  | 4978 | 8482 | 13460 | 39% | No |
| 132 | Poulton *et al.*, 1991 | KSS | 14 | 14 | 3 | 4978 | 8482 | 13460 | 40% | Yes |
| 133 | Goto *et al.*, 1990 | CPEO | 14 | 32 | 12.8 | 4978 | 8482 | 13460 | 40% | Yes |
| 134 | López-Gallardo *et al.*, 2009 | CPEO | 20 |  |  | 4978 | 8482 | 13460 | 40% | Yes |
| 135 | Chen *et al.*, 1998 | CPEO |  |  |  | 4978 | 8482 | 13460 | 44% | Yes |
| 136 | López-Gallardo *et al.*, 2009 | CPEO |  |  |  | 4978 | 8482 | 13460 | 45% | Yes |
| 137 | Wong, 2001 | KSS |  | 20 |  | 4978 | 8482 | 13460 | 45% | Yes |
| 138 | Wong, 2001 | CPEO |  | 60 |  | 4978 | 8482 | 13460 | 45% | Yes |
| 139 | Goto *et al.*, 1990 | CPEO | 12 | 15 | 18.5 | 4978 | 8482 | 13460 | 50% | Yes |
| 140 | Shoffner *et al.*, 1989 | CPEO Multisystem | 20 | 61 |  | 4978 | 8482 | 13460 | 50% | Yes |
| 141 | López-Gallardo *et al.*, 2009 | CPEO | 48 |  |  | 4978 | 8482 | 13460 | 50% | Yes |
| 142 | Wong, 2001 | KSS |  | 34 |  | 4978 | 8482 | 13460 | 51% | Yes |
| 143 | López-Gallardo *et al.*, 2009 | CPEO |  |  |  | 4978 | 8482 | 13460 | 54% | Yes |
| 144 | Gellerich 2002 |  |  |  |  | 4978 | 8482 | 13460 | 61% | No |
| 145 | López-Gallardo *et al.*, 2009 | KSS | 4 |  |  | 4978 | 8482 | 13460 | 62% | Yes |
| 146 | López-Gallardo *et al.*, 2009 | KSS |  |  |  | 4978 | 8482 | 13460 | 62% | Yes |
| 147 | Obermaier-Kusser *et al.*, 1990 | KSS | 11 | 26 |  | 4978 | 8482 | 13460 | 63% | Yes |
| 148 | Wong, 2001 | KSS |  | 16 |  | 4978 | 8482 | 13460 | 63% | Yes |
| 149 | Wong, 2001 | KSS |  | 36 |  | 4978 | 8482 | 13460 | 64% | Yes |
| 150 | Boles *et al.*, 1998 | KSS | 1 | 5 |  | 4978 | 8482 | 13460 | 65% | Yes |
| 151 | Wong, 2001 | KSS |  | 6 |  | 4978 | 8482 | 13460 | 65% | Yes |
| 152 | Gellerich 2002 |  |  |  | 48 | 4978 | 8482 | 13460 | 66% | No |
| 153 | Degoul *et al.*, 1991 | KSS | 7 | 27 |  | 4978 | 8482 | 13460 | 68% | Yes |
| 154 | López-Gallardo *et al.*, 2009 | CPEO |  |  |  | 4978 | 8482 | 13460 | 68% | Yes |
| 155 | Odoardi *et al.*, 2003 | KSS | 6 | 17 |  | 4978 | 8482 | 13460 | 69% | Yes |
| 156 | Goto *et al.*, 1990 | KSS | 10 | 14 | 41.4 | 4978 | 8482 | 13460 | 70% | Yes |
| 157 | Wong, 2001 | KSS |  | 44 |  | 4978 | 8482 | 13460 | 70% | Yes |
| 158 | López-Gallardo *et al.*, 2009 | KSS | 12 |  |  | 4978 | 8482 | 13460 | 72% | Yes |
| 159 | Ponzetto *et al.*, 1990 | KSS |  |  |  | 4978 | 8482 | 13460 | 72% | Yes |
| 160 | López-Gallardo *et al.*, 2009 | KSS |  |  |  | 4978 | 8482 | 13460 | 72% | Yes |
| 161 | Consalvo *et al.*, 1997 | KSS | 9 | 19 |  | 4978 | 8482 | 13460 | 80% | Yes |
| 162 | Degoul *et al.*, 1991 | KSS | 10 | 31 |  | 4978 | 8482 | 13460 | 80% | Yes |
| 163 | Goto *et al.*, 1990 | CPEO | 22 | 26 | 40.2 | 4978 | 8482 | 13460 | 80% | Yes |
| 164 | López-Gallardo *et al.*, 2009 | KSS |  |  |  | 4978 | 8482 | 13460 | 80% | Yes |
| 165 | Johns *et al.*, 1989 | KSS |  |  |  | 4978 | 8482 | 13460 | 83% | Yes |
| 166 | Odoardi *et al.*, 2003 | KSS | 6 | 10 |  | 4978 | 8482 | 13460 | 85% | Yes |
| 167 | Johns *et al.*, 1989 |  |  |  |  | 4978 | 8482 | 13460 | 86% | No |
| 168 | Sadikovic 2010 | CPEO + MM |  | 60 |  | 4977 | 8470 | 13447 | 6% | No |
| 169 | Sadikovic 2010 | KSS |  | 26 |  | 4977 | 8470 | 13447 | 10% | No |
| 170 | Kiyomoto *et al.*, 1997 | CPEO | 30 | 44 | 1 | 4977 | 8483 | 13460 | 14% | Yes |
| 171 | Sadikovic 2010 | Multisystem |  | 24 |  | 4977 | 8470 | 13447 | 24% | No |
| 172 | Sadikovic 2010 | Multisystem |  | 14 |  | 4977 | 8470 | 13447 | 27% | No |
| 173 | Sadikovic 2010 | CPEO + MM |  | 78 |  | 4977 | 8470 | 13447 | 27% | No |
| 174 | Sadikovic 2010 | KSS |  | 28 |  | 4977 | 8470 | 13447 | 33% | No |
| 175 | Sciacco *et al.*, 1994 | KSS |  | 35 |  | 4977 | 8470 | 13447 | 36% | Yes |
| 176 | Sadikovic 2010 | CPEO + MM |  | 39 |  | 4977 | 8470 | 13447 | 42% | No |
| 177 | Sadikovic 2010 | KSS |  | 20 |  | 4977 | 8470 | 13447 | 45% | No |
| 178 | Sadikovic 2010 | CPEO + MM |  | 60 |  | 4977 | 8470 | 13447 | 45% | No |
| 179 | Sadikovic 2010 | Multisystem |  | 45 |  | 4977 | 8470 | 13447 | 49% | No |
| 180 | Sciacco *et al.*, 1994 | CPEO | 61 | 66 |  | 4977 | 8470 | 13447 | 50% | Yes |
| 181 | Shanske *et al.*, 1990 | KSS |  |  |  | 4977 | 8470 | 13447 | 50% | Yes |
| 182 | Kiyomoto *et al.*, 1997 | CPEO | 15 | 33 | 8.5 | 4977 | 8483 | 13460 | 54% | Yes |
| 183 | Sciacco *et al.*, 1994 | KSS | 11 | 13 |  | 4977 | 8470 | 13447 | 55% | Yes |
| 184 | Sadikovic 2010 | CPEO + MM |  | 56 |  | 4977 | 8470 | 13447 | 55% | No |
| 185 | Sadikovic 2010 | CPEO |  | 39 |  | 4977 | 8470 | 13447 | 60% | No |
| 186 | Sadikovic 2010 | KSS |  | 13 |  | 4977 | 8470 | 13447 | 61% | No |
| 187 | Sadikovic 2010 | KSS |  | 16 |  | 4977 | 8470 | 13447 | 62% | No |
| 188 | Sudoyo *et al.*, 1993 | CPEO | 4 | 41 |  | 4977 | 8470 | 13447 | 64% | Yes |
| 189 | Sadikovic 2010 | MM |  | 36 |  | 4977 | 8470 | 13447 | 64% | No |
| 190 | Sadikovic 2010 | Multisystem |  | 6 |  | 4977 | 8470 | 13447 | 65% | No |
| 191 | Sadikovic 2010 | Renal tubular acidosis |  | 8 |  | 4977 | 8470 | 13447 | 67% | No |
| 192 | Sadikovic 2010 | CPEO + MM |  | 9 |  | 4977 | 8470 | 13447 | 67% | No |
| 193 | Sadikovic 2010 | CPEO + MM |  | 36 |  | 4977 | 8470 | 13447 | 70% | No |
| 194 | Sadikovic 2010 | KSS |  | 44 |  | 4977 | 8470 | 13447 | 70% | No |
| 195 | Sciacco *et al.*, 1994 | KSS |  | 28 |  | 4977 | 8470 | 13447 | 75% | Yes |
| 196 | Kiyomoto *et al.*, 1997 | CPEO Multisystem | 12 | 14 | 18 | 4977 | 8483 | 13460 | 77% | Yes |
| 197 | Mita 1989 | KSS |  | 30 | 51 | 4977 | 8483 | 13460 | 80% | No |
| 198 | Sadikovic 2010 | CPEO + MM |  | 27 |  | 4977 | 8470 | 13447 | 85% | No |
| 199 | Sadikovic 2010 | PEO |  | 20 |  | 4977 | 8470 | 13447 | 88% | No |
| 200 | Solano *et al.*, 2003 | CPEO |  |  |  | 4958 | 8380 | 13338 | 30% | Yes |
| 201 | Pistilli *et al.*, 2003 | KSS | 10 | 36 |  | 4949 | 8631 | 13580 | 60% | Yes |
| 202 | Oldfors 1992 |  |  | 27 | 20 | 4914 | 7586 | 12500 | 79% | No |
| 203 | Barrientos *et al.*, 1995 | KSS | 3 | 28 |  | 4800 |  |  | 38% | Yes |
| 204 | Larsson and Holme, 1992 | KSS | 5 | 12 |  | 4800 | 10800 | 15600 | 87% | No |
| 205 | López-Gallardo *et al.*, 2009 | KSS |  |  |  | 4754 | 11292 | 16046 | 45% | Yes |
| 206 | Oldfors 1992 |  |  | 11 | 1 | 4700 | 10500 | 15200 | 87% | No |
| 207 | Degoul *et al.*, 1991 | KSS | 18 | 25 |  | 4500 | 11300 | 15800 | 30% | Yes |
| 208 | Johns *et al.*, 1989 | CPEO |  |  |  | 4500 | 9300 | 13800 | 55% | Yes |
| 209 | Zeviani *et al.*, 1988 | KSS | 14 | 26 |  | 4500 | 9000 | 13500 | 62% | Yes |
| 210 | Zeviani *et al.*, 1988 | KSS | 14 | 28 |  | 4500 | 9000 | 13500 | 75% | Yes |
| 211 | Solano *et al.*, 2003 | KSS | 7 | 18 |  | 4421 | 10951 | 15372 | 80% | Yes |
| 212 | Kiyomoto *et al.*, 1997 | KSS | 4 | 15 | 3.5 | 4420 | 10952 | 15372 | 20% | Yes |
| 213 | Sadikovic 2010 | Multisystem |  | 8 |  | 4420 | 10560 | 14980 | 55% | No |
| 214 | Wong, 2001 | KSS |  | 8 |  | 4420 | 10560 | 14980 | 55% | Yes |
| 215 | Sadikovic 2010 | Multisystem |  | 48 |  | 4369 | 9256 | 13625 | 38% | No |
| 216 | Carod-Artal *et al.*, 2003 | CPEO | 19 | 26 |  | 4238 | 9500 | 13738 | 55% | Yes |
| 217 | Solano *et al.*, 2003 | CPEO |  |  |  | 4238 | 9485 | 13723 | 55% | No |
| 218 | Goto *et al.*, 1990 | CPEO | 40 | 52 | 19 | 4200 | 5850 | 10050 | 30% | Yes |
| 219 | Zeviani *et al.*, 1988 | KSS | 17 | 27 |  | 4200 | 9000 | 13200 | 66% | Yes |
| 220 | Pineda *et al.*, 2006 | KSS | 7 | 8 |  | 4124 | 11033 | 15157 | 72% | Yes |
| 221 | Blakely *et al.*, 2004 | CPEO |  |  | 20 | 4115 | 11262 | 15375 | 66% | Yes |
| 222 | Gellerich 2002 |  |  |  |  | 4093 | 10057 | 14150 | 62% | No |
| 223 | De Coo *et al.*, 1997 | KSS |  |  |  | 4000 |  |  | 60% | Yes |
| 224 | Kiyomoto *et al.*, 2006 | CPEO | 60 | 62 | 14 | 3800 |  |  | 4% | Yes |
| 225 | Vielhaber *et al.*, 2002 | CPEO |  | 48 |  | 3800 |  |  | 10% | Yes |
| 226 | Kornblum *et al.*, 2004 | CPEO | 28 | 35 |  | 3800 |  |  | 45% | Yes |
| 227 | Solano *et al.*, 2003 | KSS | 14 | 31 |  | 3720 | 11727 | 15447 | 50% | Yes |
| 228 | Kiyomoto *et al.*, 1997 | CPEO | 27 | 43 | 8 | 3716 | 10845 | 14561 | 44% | Yes |
| 229 | Kiyomoto *et al.*, 2006 | CPEO | 18 | 28 | 12 | 3700 |  |  | 65% | Yes |
| 230 | Degoul *et al.*, 1991 | CPEO | 31 | 51 |  | 3513 | 7483 | 10996 | 40% | Yes |
| 231 | Wong, 2001 |  |  |  |  | 3500 |  |  | 16% | No |
| 232 | Gellerich 2002 |  |  |  |  | 3500 | 11300 | 14800 | 58% | No |
| 233 | Kornblum *et al.*, 2005a | KSS | 20 | 37 |  | 3500 |  |  | 62% | Yes |
| 234 | Schroder *et al.*, 2000 | KSS |  | 30 |  | 3500 |  |  | 62% | Yes |
| 235 | Schroder *et al.*, 2000 | CPEO |  | 29 |  | 3500 |  |  | 65% | Yes |
| 236 | Lertrit *et al.*, 1999 | KSS | 35 | 37 |  | 3485 | 10280 | 13765 | 37% | Yes |
| 237 | Barbiroli *et al.*, 1995 | CPEO | 22 | 30 |  | 3300 |  |  | 20% | Yes |
| 238 | Wong *et al.*, 2003 | KSS | 29 | 36 |  | 3079 | 8419 | 11498 | 92% | Yes |
| 239 | Sadikovic 2010 | Multisystem |  | 25 |  | 3030 | 10958 | 13988 | 47% | No |
| 240 | Sadikovic 2010 | KSS |  | 41 |  | 2976 | 8388 | 11364 | 92% | No |
| 241 | Goto *et al.*, 1990 | CPEO | 35 | 45 | 16.6 | 2800 | 11500 | 14300 | 50% | Yes |
| 242 | Kornblum *et al.*, 2005a | CPEO Multisystem | 44 | 49 |  | 2700 |  |  | 32% | Yes |
| 243 | Gellerich 2002 |  |  |  | 22 | 2600 | 11000 | 13600 | 62% | No |
| 244 | Kornblum *et al.*, 2005b | CPEO | 48 | 48 |  | 2500 |  |  | 39% | Yes |
| 245 | Ohno *et al.*, 1996 | KSS | 26 | 27 |  | 2500 |  |  | 88% | Yes |
| 246 | Solano *et al.*, 2003 | KSS |  |  |  | 2434 | 10620 | 13054 | 77% | Yes |
| 247 | Solano *et al.*, 2003 | KSS |  |  |  | 2310 | 12112 | 14422 | 70% | Yes |
| 248 | Kiyomoto *et al.*, 1997 | CPEO | 20 | 47 | 20 | 2309 | 12113 | 14422 | 59% | Yes |
| 249 | Kiyomoto *et al.*, 1997 | CPEO | 21 | 31 | 4 | 2309 | 12113 | 14422 | 89% | Yes |
| 250 | Goto *et al.*, 1990 | CPEO | 39 | 70 | 10.7 | 2300 | 11000 | 13300 | 50% | Yes |
| 251 | Moraes *et al.*, 1992 | CPEO |  |  |  | 2300 |  |  | 80% | Yes |
| 252 | Goto *et al.*, 1990 | CPEO | 34 | 37 | 7.2 | 2200 | 12000 | 14200 | 50% | Yes |
| 253 | Goto *et al.*, 1990 | CPEO | 52 | 55 | 19.3 | 2200 | 12000 | 14200 | 60% | Yes |
| 254 | Zeviani *et al.*, 1988 | KSS | 24 | 33 |  | 2060 | 7440 | 9500 | 60% | Yes |
| 255 | Schroder *et al.*, 2000 | CPEO |  | 45 |  | 2000 |  |  | 32% | Yes |
| 256 | Goto *et al.*, 1990 | KSS | 35 | 36 | 12.6 | 1800 | 13000 | 14800 | 50% | Yes |

**Supplementary Table 3** **Cases excluded from the meta-analysis.**

Details of the excluded case and reasons for exclusion are noted.

| **Study** | **Pheno-**  **type** | **Age at Onset** | **Age at Biopsy** | **COX**  **%** | **Deletion**  **Size** | **Break**  **point 3’** | **Break**  **point 5’** | **Het%** | **Notes** | **In López-Gallardo *et al.*, 2009** |
| --- | --- | --- | --- | --- | --- | --- | --- | --- | --- | --- |
| Mori *et al.*, 1991 | KSS | 2 | 6 |  | 6596 | 6383 | 12979 | 60% | Deletion size does not match those in the original publication | yes |
| Larsson and Holme, 1992 | KSS | 16 | 26 |  | 4667 | 7697 | 12364 | 70% | Deletion size does not match those in the original publication | yes |
| Goto *et al.*, 1990 | CPEO | 13 | 31 | 50% | 5300 | 9206 | 14506 | 90% | Deletion size is inconsistent with reported breakpoints | yes |
| Schaefer *et al.*, 2005 | CPEO | 15 | 24 |  | 7400 |  |  | 8% | In our cohort | yes |
| Zoccolella *et al.*, 2006 |  | 6 | 32 |  | 1813 | 3505 | 5318 | 70% | Location is unlike any other deletion in the study, excluded as an outlier | yes |
| Reynier *et al.*, 1994 | CPEO |  | 41 |  |  |  |  | 50% | Other mutations; also contains a point mutation | yes |
| Odoardi *et al.*, 2003 | KSS | 20 | 27 |  | 4978 | 8482 | 13460 | 51% | Other mutations; Dimers | yes |
| Odoardi *et al.*, 2003 | CPEO | 30 | 40 |  | 7000 |  |  | 51% | Other mutations; Dimers | yes |
| Brockington *et al.*, 1995 | KSS | 12 | 19 |  | 8562 | 7354 | 15916 | 27% | Other mutations; Dimers and duplications | yes |
| Brockington *et al.*, 1995 | KSS | 4 | 22 |  | 4978 | 8482 | 13460 | 89% | Other mutations; Dimers and duplications | yes |
| Jacobs *et al.*, 2004 | PS/KSS | 0 | 3 |  | 8034 | 7934 | 15968 |  | Other mutations; Dimers and duplications | no |
| Jacobs *et al.*, 2004 | PS | 0 | 1.5 |  | 3444 | 6097 | 9541 | 64% | Other mutations; Dimers and duplications | no |
| Tengan *et al.*, 1998 | KSS | 5 | 12 |  | 9660 | 5784 | 15444 | 65% | Other mutations; Duplications | yes |
| Vazquez-Acevedo *et al.*, 2002 | KSS | 8 | 23 |  | 4978 | 8482 | 13460 | 75% | Other mutations; multiple deletions | yes |
| Tanaka *et al.*, 1989 | CPEO | 39 | 70 |  | 2100 |  |  | 36% | Uncertain breakpoints | yes |
| Oldfors 1992 |  |  | 52 | 10% | 3940 | 10060 | 14000 | 76% | Uncertain breakpoints | no |
| Kunz *et al.*, 1997 | CPEO |  | 34 |  | 3200 |  |  | 53% | Uncertain breakpoints | yes |
| Kunz *et al.*, 1997 | CPEO |  | 55 |  | 4700 |  |  | 67% | Uncertain breakpoints | yes |
| Kunz *et al.*, 1997 | CPEO |  | 32 |  | 2600 |  |  | 84% | Uncertain breakpoints | yes |
| Marzuki *et al.*, 1997 | CPEO | 20 | 59 |  | 5020 | 9780 | 14800 | 16% | Uncertain breakpoints and multiple deletions | yes |
| Sudoyo *et al.*, 1993 | CPEO | 25 | 60 |  | 2664 | 12336 | 15000 | 55% | Uncertain breakpoints and multiple deletions | yes |

# References

Barbiroli, B., *et al.*, 1995. Lipoic (thioctic) acid increases brain energy availability and skeletal muscle performance as shown by in vivo 31P-MRS in a patient with mitochondrial cytopathy. J Neurol. 242**,** 472-7.

Barrientos, A., *et al.*, 1995. [Progressive external ophthalmoplegia and the Kearns-Sayre syndrome: a clinical and molecular study of 6 cases]. Med Clin (Barc). 105**,** 180-4.

Bernes, S.M., *et al.*, 1993. Identical mitochondrial DNA deletion in mother with progressive external ophthalmoplegia and son with Pearson marrow-pancreas syndrome. J Pediatr. 123**,** 598-602.

Blakely, E.L., *et al.*, 2004. Mitochondrial DNA deletion in "identical" twin brothers. J Med Genet. 41**,** e19.

Blok, R.B., *et al.*, 1995. A topoisomerase II cleavage site is associated with a novel mitochondrial DNA deletion. Hum Genet. 95**,** 75-81.

Boles, R.G., *et al.*, 1998. Mitochondrial DNA deletion with Kearns Sayre syndrome in a child with Addison disease. Eur J Pediatr. 157**,** 643-7.

Brockington, M., *et al.*, 1995. Kearns-Sayre syndrome associated with mitochondrial DNA deletion or duplication: a molecular genetic and pathological study. J Neurol Sci. 131**,** 78-87.

Carod-Artal, F.J., *et al.*, 2003. A single deletion of mitochondrial DNA in a Brazilian patient with chronic progressive external ophthalmoplegia. Deleción en el ADN mitocondrial de una paciente Brasileña con oftalmoplejía crónica progresiva externa. 37**,** 1029-1031.

Carta, A., *et al.*, 2000. Ultrastructural analysis of extraocular muscle in chronic progressive external ophthalmoplegia. Arch Ophthalmol. 118**,** 1441-5.

Chen, Q., *et al.*, 1998. Mitochondrial gene defect in patients with chronic progressive external ophthalmoplegia. Chin Med J (Engl). 111**,** 500-3.

Consalvo, D., *et al.*, 1997. [Severe cardiac failure in Kearns-Sayre syndrome]. Medicina (B Aires). 57**,** 67-71.

De Coo, I.F.M., *et al.*, 1997. A PCR test for progressive external ophthalmoplegia and Kearns-Sayre syndrome on DNA from blood samples. Journal of the Neurological Sciences. 149**,** 37-40.

Degoul, F., *et al.*, 1991. Deletions of mitochondrial DNA in Kearns-Sayre syndrome and ocular myopathies: genetic, biochemical and morphological studies. J Neurol Sci. 101**,** 168-77.

Emma, F., *et al.*, 2006. "Bartter-like" phenotype in Kearns-Sayre syndrome. Pediatr Nephrol. 21**,** 355-60.

Fromenty, B., *et al.*, 1996. Efficient and specific amplification of identified partial duplications of human mitochondrial DNA by long PCR. Biochim Biophys Acta. 1308**,** 222-30.

Gellerich, F.N., *et al.*, 2002. Mitochondrial respiratory rates and activities of respiratory chain complexes correlate linearly with heteroplasmy of deleted mtDNA without threshold and independently of deletion size. Biochim Biophys Acta. 1556**,** 41-52.

Goto, Y., *et al.*, 1990. Chronic progressive external ophthalmoplegia: a correlative study of mitochondrial DNA deletions and their phenotypic expression in muscle biopsies. J Neurol Sci. 100**,** 63-9.

Heddi, A., *et al.*, 1994. Steady state levels of mitochondrial and nuclear oxidative phosphorylation transcripts in Kearns-Sayre syndrome. Biochim Biophys Acta. 1226**,** 206-12.

Ishikawa, Y., Goto, Y.I., Minami, R., 2000. Progression in a case of Kearns-Sayre syndrome. Journal of Child Neurology. 15**,** 750-755.

Jacobs, L.J., *et al.*, 2004. Pearson syndrome and the role of deletion dimers and duplications in the mtDNA. J Inherit Metab Dis. 27**,** 47-55.

Johns, D.R., Hurko, O., 1989. Preferential amplification and molecular characterization of junction sequences of a pathogenetic deletion in human mitochondrial DNA. Genomics. 5**,** 623-8.

Johns, D.R., *et al.*, 1989. Directly repeated sequences associated with pathogenic mitochondrial DNA deletions. Proc Natl Acad Sci U S A. 86**,** 8059-62.

Kiyomoto, B.H., *et al.*, 1997. Mitochondrial DNA defects in Brazilian patients with chronic progressive external ophthalmoplegia. Journal of the Neurological Sciences. 152**,** 160-165.

Kiyomoto, B.H., *et al.*, 2006. Frequency of dystrophic muscle abnormalities in chronic progressive external ophthalmoplegia: analysis of 86 patients. J Neurol Neurosurg Psychiatry. 77**,** 541-3.

Kornblum, C., *et al.*, 2004. [Diagnostic value of mitochondrial DNA analysis in chronic progressive external ophthalmoplegia (CPEO)]. Klin Monbl Augenheilkd. 221**,** 1057-61.

Kornblum, C., *et al.*, 2005a. Sensorineural hearing loss in patients with chronic progressive external ophthalmoplegia or Kearns-Sayre syndrome. J Neurol. 252**,** 1101-7.

Kornblum, C., *et al.*, 2005b. Creatine has no beneficial effect on skeletal muscle energy metabolism in patients with single mitochondrial DNA deletions: a placebo-controlled, double-blind 31P-MRS crossover study. European Journal of Neurology. 12**,** 300-309.

Kunz, W.S., *et al.*, 1997. Detection of mitochondrial defects by laser fluorimetry. Mol Cell Biochem. 174**,** 97-100.

Larsson, N.G., Holme, E., 1992. Multiple short direct repeats associated with single mtDNA deletions. Biochim Biophys Acta. 1139**,** 311-4.

Lertrit, P., *et al.*, 1999. A unique 3.5-kb deletion of the mitochondrial genome in Thai patients with Kearns-Sayre syndrome. Human Genetics. 105**,** 127-131.

López-Gallardo, E., *et al.*, 2009. CPEO and KSS differ in the percentage and location of the mtDNA deletion. Mitochondrion. 9**,** 314-317.

Marie, S.K., *et al.*, 1999. Kearns-Sayre syndrome "plus". Classical clinical findings and dystonia. Arq Neuropsiquiatr. 57**,** 1017-23.

Marin-Garcia, J., *et al.*, 2002. Severe mitochondrial cytopathy with complete A-V block, PEO, and mtDNA deletions. Pediatr Neurol. 27**,** 213-6.

Marzuki, S., *et al.*, 1997. Developmental genetics of deleted mtDNA in mitochondrial oculomyopathy. J Neurol Sci. 145**,** 155-62.

Matsuoka, T., *et al.*, 1992. Segmental cytochrome c-oxidase deficiency in CPEO: teased muscle fiber analysis. Muscle Nerve. 15**,** 209-13.

Mita, S., *et al.*, 1989. Detection of 'deleted' mitochondrial genomes in cytochrome-c oxidase-deficient muscle fibers of a patient with Kearns-Sayre syndrome. Proceedings of the National Academy of Sciences of the United States of America. 86**,** 9509-9513.

Montiel-Sosa, J.F., *et al.*, 2013. Phylogenetic analysis of mitochondrial DNA in a patient with Kearns-Sayre syndrome containing a novel 7629-bp deletion. Mitochondrial DNA.

Moraes, C.T., *et al.*, 1992. Molecular analysis of the muscle pathology associated with mitochondrial DNA deletions. Nature Genetics. 1**,** 359-367.

Mori, K., *et al.*, 1991. Renal and skin involvement in a patient with complete Kearns-Sayre syndrome. Am J Med Genet. 38**,** 583-7.

Obermaier-Kusser, B., *et al.*, 1990. Different copy numbers of apparently identically deleted mitochondrial DNA in tissues from a patient with Kearns-Sayre syndrome detected by PCR. Biochem Biophys Res Commun. 169**,** 1007-15.

Odoardi, F., *et al.*, 2003. Pathogenic role of mtDNA duplications in mitochondrial diseases associated with mtDNA deletions. Am J Med Genet A. 118A**,** 247-54.

Ohno, K., *et al.*, 1996. MELAS- and Kearns-Sayre-type co-mutation [corrected] with myopathy and autoimmune polyendocrinopathy. Ann Neurol. 39**,** 761-6.

Okulla, T., *et al.*, 2005. Diagnostic value of mitochondrial DNA mutation analysis in juvenile unilateral ptosis. Graefes Arch Clin Exp Ophthalmol. 243**,** 380-2.

Oldfors, A., *et al.*, 1992. Mitochondrial DNA deletions and cytochrome c oxidase deficiency in muscle fibers. Journal of the Neurological Sciences. 110**,** 169-177.

Pineda, M., *et al.*, 2004. Familiar chronic progressive external ophthalmoplegia of mitochondrial origin. Oftalmoplejía crónica progresiva externa familiar de origen mitocondrial. 38**,** 1023-1027.

Pineda, M., *et al.*, 2006. Cerebral folate deficiency and leukoencephalopathy caused by a mitochondrial DNA deletion. Annals of Neurology. 59**,** 394-398.

Pistilli, D., *et al.*, 2003. Detection of deleted mitochondrial DNA in Kearns-Sayre syndrome using laser capture microdissection. Hum Pathol. 34**,** 1058-61.

Ponzetto, C., *et al.*, 1990. Kearns-Sayre syndrome: different amounts of deleted mitochondrial DNA are present in several autoptic tissues. J Neurol Sci. 96**,** 207-10.

Poulton, J., *et al.*, 1991. Germ-line deletions of mtDNA in mitochondrial myopathy. Am J Hum Genet. 48**,** 649-53.

Reynier, P., *et al.*, 1994. Association of deletion and homoplasmic point mutation of the mitochondrial DNA in an ocular myopathy. Biochem Biophys Res Commun. 202**,** 1606-11.

Sadikovic, B., *et al.*, 2010. Sequence Homology at the Breakpoint and Clinical Phenotype of Mitochondrial DNA Deletion Syndromes. PLoS One. 5**,** e15687.

Schaefer, A.M., *et al.*, 2005. Ophthalmoplegia due to mitochondrial DNA disease: the need for genetic diagnosis. Muscle Nerve. 32**,** 104-7.

Schröder, R., *et al.*, 2000. New insights into the metabolic consequences of large-scale mtDNA deletions: A quantitative analysis of biochemical, morphological, and genetic findings in human skeletal muscle. Journal of Neuropathology and Experimental Neurology. 59**,** 353-360.

Sciacco, M., *et al.*, 1994. Distribution of wild-type and common deletion forms of mtDNA in normal and respiration-deficient muscle fibers from patients with mitochondrial myopathy. Human Molecular Genetics. 3**,** 13-19.

Shanske, S., *et al.*, 1990. Widespread tissue distribution of mitochondrial DNA deletions in Kearns-Sayre syndrome. Neurology. 40**,** 24-8.

Shanske, S., *et al.*, 2002. Identical mitochondrial DNA deletion in a woman with ocular myopathy and in her son with pearson syndrome. Am J Hum Genet. 71**,** 679-83.

Shoffner, J.M., *et al.*, 1989. Spontaneous Kearns-Sayre/chronic external ophthalmoplegia plus syndrome associated with a mitochondrial DNA deletion: A slip-replication model and metabolic therapy. Proceedings of the National Academy of Sciences of the United States of America. 86**,** 7952-7956.

Simaan, E.M., *et al.*, 1999. Unusual presentation of Kearns-Sayre syndrome in early childhood. Pediatr Neurol. 21**,** 830-1.

Solano, A., *et al.*, 2003. Characterisation of repeat and palindrome elements in patients harbouring single deletions of mitochondrial DNA. Journal of medical genetics. 40.

Sudoyo, H., *et al.*, 1993. Phenotypic expression of mtDNA heteroplasmy in the skeletal muscle of patients with oculomyopathy: defect in mitochondrial protein synthesis. J Neurol Sci. 117**,** 83-91.

Tanaka, M., *et al.*, 1989. Differently deleted mitochondrial genomes in maternally inherited chronic progressive external ophthalmoplegia. J Inherit Metab Dis. 12**,** 359-62.

Tengan, C.H., *et al.*, 1998. Mitochondrial encephalomyopathy and hypoparathyroidism associated with a duplication and a deletion of mitochondrial deoxyribonucleic acid. J Clin Endocrinol Metab. 83**,** 125-9.

Vazquez-Acevedo, M., *et al.*, 1995. Characterization of a 5025 base pair mitochondrial DNA deletion in Kearns-Sayre syndrome. Biochim Biophys Acta. 1271**,** 363-8.

Vazquez-Acevedo, M., *et al.*, 2002. A case of Kearns-Sayre syndrome with the 4,977-bp common deletion associated with a novel 7,704-bp deletion. Neurol Sci. 23**,** 247-50.

Vielhaber, S., *et al.*, 2002. Expression pattern of mitochondrial respiratory chain enzymes in skeletal muscle of patients harboring the A3243G point mutation or large-scale deletions of mitochondrial DNA. J Neuropathol Exp Neurol. 61**,** 885-95.

Wong, L.J., 2001. Recognition of mitochondrial DNA deletion syndrome with non-neuromuscular multisystemic manifestation. Genet Med. 3**,** 399-404.

Wong, L.J., *et al.*, 2003. Compensatory amplification of mtDNA in a patient with a novel deletion/duplication and high mutant load. J Med Genet. 40**,** e125.

Zeviani, M., *et al.*, 1988. Deletions of mitochondrial DNA in Kearns-Sayre syndrome. Neurology. 38**,** 1339-46.

Zoccolella, S., *et al.*, 2006. Unusual clinical presentation of a patient carrying a novel single 1.8 kb deletion of mitochondrial DNA. Funct Neurol. 21**,** 39-41.
